# Investigation of Novel Regulation of N-myristoyltransferase by Mammalian Target of Rapamycin in Breast Cancer Cells

**DOI:** 10.1038/s41598-018-30447-0

**Published:** 2018-08-28

**Authors:** Marine Jacquier, Shiby Kuriakose, Apurva Bhardwaj, Yang Zhang, Anuraag Shrivastav, Stéphanie Portet, Shailly Varma Shrivastav

**Affiliations:** 1 Department of Mathematics, University of Manitoba, Winnipeg, Canada; 2 Department of Biology, University of Winnipeg, Winnipeg, Manitoba Canada; 3 Department of Biochemistry and Medical Genetics, University of Manitoba, Winnipeg, Canada; 4 Research Institute of Hematology and Oncology, CancerCare Manitoba, Winnipeg, Manitoba Canada

## Abstract

Breast cancer is the most common cancer in women worldwide. Hormone receptor breast cancers are the most common ones and, about 2 out of every 3 cases of breast cancer are estrogen receptor (ER) positive. Selective ER modulators, such as tamoxifen, are the first line of endocrine treatment of breast cancer. Despite the expression of hormone receptors some patients develop tamoxifen resistance and 50% present de novo tamoxifen resistance. Recently, we have demonstrated that activated mammalian target of rapamycin (mTOR) is positively associated with overall survival and recurrence free survival in ER positive breast cancer patients who were later treated with tamoxifen. Since altered expression of protein kinase B (PKB)/Akt in breast cancer cells affect N-myristoyltransferase 1 (NMT1) expression and activity, we investigated whether mTOR, a downstream target of PKB/Akt, regulates NMT1 in ER positive breast cancer cells (MCF7 cells). We inhibited mTOR by treating MCF7 cells with rapamycin and observed that the expression of NMT1 increased with rapamycin treatment over the period of time with a concomitant decrease in mTOR phosphorylation. We further employed mathematical modelling to investigate hitherto not known relationship of mTOR with NMT1. We report here for the first time a collection of models and data validating regulation of NMT1 by mTOR.

## Introduction

The hormone receptor status in breast cancer (BC) is crucial for deciding treatment regimen for BC patients. The presence of estrogen receptor (ER) predicts treatment response to endocrine therapy, primarily due to its role in driving ER positive breast cancer cells to proliferate^1^. However, it has been observed that at least 50% of ER positive tumors display de novo resistance to endocrine therapies such as tamoxifen, and many of those initially sensitive acquire resistance despite expressing non-mutated ER^2^. Earlier studies suggest activation of mTOR potentially plays a role in endocrine resistance^3,4^. Recently we demonstrated that activated mTOR (as measured by phosphorylation at serine (S) 2448 residue) in treatment naive breast tumors is positively associated with overall survival (OS) and recurrence free survival (RFS) in ER positive breast cancer patients who were later treated with tamoxifen^5^. Also, we demonstrated that ER is a substrate of mTOR and interacts with it further supporting the crosstalk between ER and mTOR. Therefore, we concluded that in breast tumors where there is an intact estrogen regulated mTOR signaling, mTOR is associated with an increased likelihood of responsiveness to endocrine therapy^5^. Furthermore, very recently, we observed that N-myristoyltransferase (NMT1) is a downstream target of mTOR (Jaksic *et al*. 2018 unpublished data).

mTOR is a serine/threonine kinase that regulates cell growth, proliferation, motility and survival^6^. It is a component of the phosphatidylinositol 3-kinase (PI3K) cell survival pathway and operates at a key junction in the PI3K pathway as it acts both upstream as well as downstream of protein kinase B (PKB or Akt)^6^. As a signaling hub, mTOR exists in two different multiprotein complexes: mTORC1 and mTORC2 that are involved in cell growth^7,8^. mTORC1 is mainly responsible for the regulation of protein synthesis necessary for cell growth and proliferation^9,10^, whereas mTORC2 in a feedback loop manner is responsible for the phosphorylation of Akt at S473^11^. mTORC1 is mainly activated by PI3K/Akt pathway whereas inhibited by the tuberous sclerosis 1/2 (TSC1/TSC2) complex^12^. Once the mTORC1 is active it can exert numerous biological effects by phosphorylating the downstream targets.

Rapamycin, produced by the bacterium *Streptomyces hygroscopicus*, was identified as an anti-fungal agent^13^. Rapamycin and its derivatives have been used as therapeutic agents with immunosuppressant and anti-tumor properties. The action of rapamycin is mediated by the specific inhibition of mTOR protein kinase. Rapamycin and its analogues are first generation mTOR inhibitors and have been used in human tumors as monotherapy or as a component of combination therapy. Activation of various growth factor receptors such as human epidermal growth factor receptor 2 (HER-2) and insulin like growth factor receptor (IGFR) results in dysregulation of PI3K/Akt signaling. Dysregulation of various components of mTOR signaling pathway has been reported in various cancers such as breast, ovarian, renal, colon and head and neck cancers. Activation of Akt-mTOR pathway is also associated with the initiation of melanocyte tumors^14^. In conclusion, mTOR has been suggested to play a key role in the oncogenic process. mTOR is a downstream target of EGFR signaling and therefore considered as an important therapeutically attractive target for the treatment of various types of cancer.

Myristoylation is a co-translational lipid modification that involves the attachment of a 14-carbon saturated fatty acid or myristate to the N-terminal glycine residue of a subset of eukaryotic proteins^15,16^. NMT belongs to GCN5-related N-acetyltransferases superfamily of proteins and catalyzes myrisate transfer^17,18^. NMT has been isolated and characterized in yeast, fungi, protozoan parasites, plants and mammals including mouse, rat and human^19–22^. Studies have shown that most of the mammalian species including humans possess two NMT enzymes that are encoded by distinct genes^23^. NMT1 and NMT2 in humans share 77% amino acid sequence identity and have similar substrate affinities^23,24^. Human NMT1 and NMT2 are homologous to mouse and rat. However, human and mouse versions of NMT1 and NMT2 are highly homologous and share more than 95% amino acid sequence identity^23^. Myristoylation is an integral part of apoptosis and the myristoylated proteins have been reported to be involved in various cellular processes including cellular proliferation and oncogenesis^25–29^. Some examples of myristoylated proteins are the catalytic subunit of cAMP-dependent protein kinase, *β*-subunit of calcineurin, *α*-subunit of several G-proteins, several tyrosine kinases, etc^30^. Evidence from various studies have suggested the involvement of NMT1 in cancer^26^. The altered expressions of NMT1 are observed in different types of cancer such as colon, breast, gallbladder and brain^26^. NMT activity and expression has been reported to be up-regulated during the progression of colorectal cancer^31^. Strong expression of NMT has been reported in malignant breast tissues compared with normal breast cells suggesting that NMT could be an important player in breast cancer^29^.

Very recently, we observed that NMT1 is a downstream target of mTOR (Jaksic *et al*., 2018 unpublished data). In this work, we investigated the regulation of NMT1 by mTOR and further determined the impact of perturbations such as the effect of drugs by combining *in vitro* experiments and mathematical modelling approaches. We treated ER positive breast cancer cells with rapamycin and determined the effect of mTOR inhibition on NMT1 in a time dependent manner. Signaling pathways involving mTOR have not been extensively studied mathematically or computationally^32–35^. Most models of mTOR pathway computationally investigate the signaling upstream of mTOR, in particular, the relationship between insulin signaling and mTOR. The complexity of these models is variable, from a few molecules to dozens, allowing to investigate the outcome of potential signaling events, in order to have a better knowledge of the pathway and/or to determine the impact of perturbations such as the effect of drugs^32,33,36–38^. To the best of our knowledge, the regulation of NMT1 by mTOR has never been mathematically modelled. In this study, we propose a collection of models of this regulation, including the inhibition of mTOR by rapamycin. The use of a collection of models allowed us to consider a variety of assumptions on the endogenous level of mTOR, the feedback regulation of mTOR by NMT1 and characteristics of the pathway when perturbed by rapamycin. All models were calibrated and validated by fitting their responses to experimental data; then, the best models were identified. Confronting models’ predictions to experimental data will help us determine key characteristics that are difficult to obtain experimentally, such as the relevance of the negative feedback of NMT1 on mTOR and the reversibility of the inhibition of mTOR by rapamycin.

## Results

Rapamycin acts as an inhibitor of mTOR and inhibits phosphorylation at S2448 residue of mTOR. In this study, we investigated the effects of rapamycin treatment on the expression of total NMT1 over time.

### Rapamycin augments NMT1 expression

**Figure 1:**
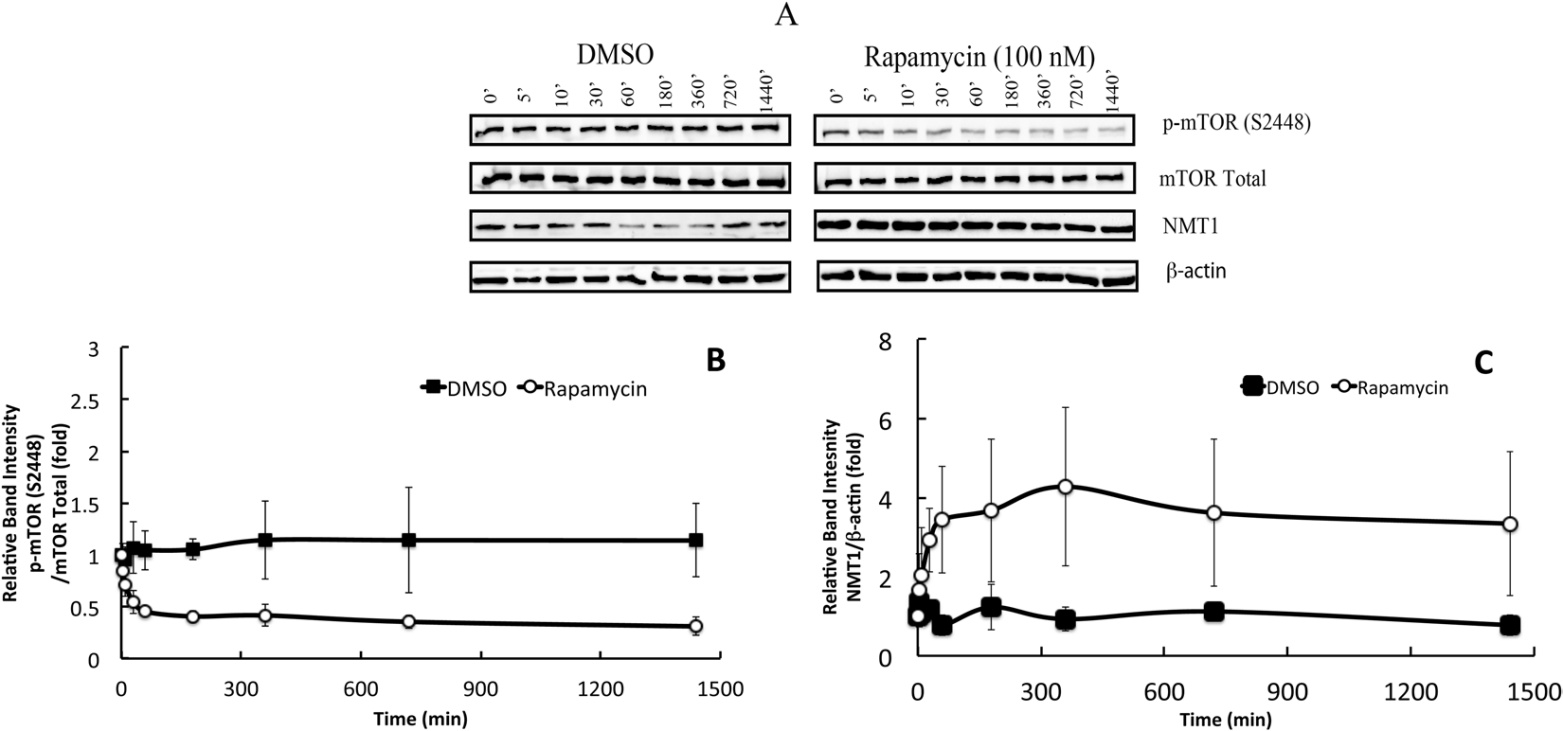
Rapamycin treatment decreased the phosphorylation of mTOR (S2448) and augmented NMT1 levels in a time-dependent manner. MCF7 cells at 70–80% confluence were starved and treated with rapamycin (100 nM) or DMSO for indicated time points and protein lysates were prepared as mentioned in the materials and methods section. (**A**) A representative Western blot analysis displaying the expression levels of p-mTOR (S2448), mTOR total, NMT1 and *β*-actin. The depiction of relative band intensities of p-mTOR (S2448)/total mTOR (**B**) and NMT1/*β*-actin (**C**) when MCF7 cells were treated with rapamycin or DMSO for indicated time points. All the experimental data were normalized against control 0′ (no treatment) which is 1-fold. The data point is an average of four independent experiments and the bars represent the standard deviation between them.

**Figure 2:**
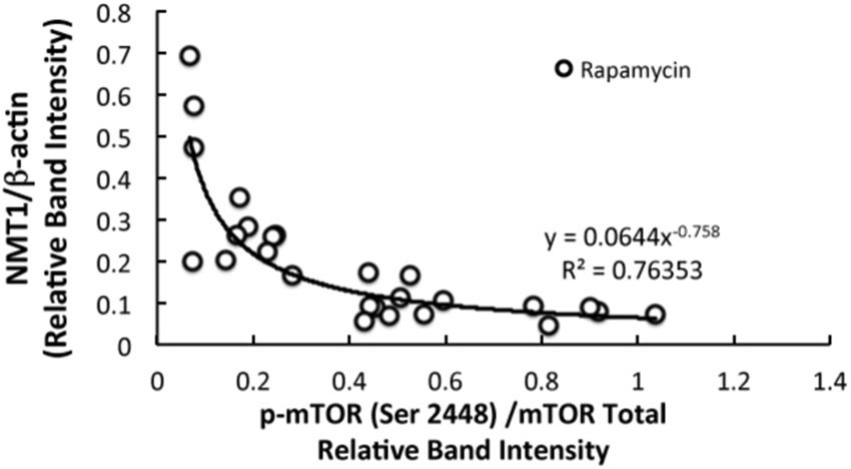
Correlation between active mTOR and NMT-1 in rapamycin treated MCF7 cells. Relative band intensities of p-mTOR (S2448), total mTOR, NMT1 and *β*-actin were obtained from Western blot analyses. Active mTOR is the ratio of relative band intensities of p-mTOR(S2448)/mTOR Total and NMT1 is the ratio of relative band intensities of NMT1/*β*-actin.

The MCF7 cells were treated with either 100 nM rapamycin or an equivalent volume of DMSO (10 *μ*L) for 5′, 10′, 30′, 60′, 180′, 360′, 720′and 1440′ following which cell lysates were collected and subjected to Western blot analyses as indicated in the Materials and Methods section. Rapamycin treated MCF7 cells showed a decrease in the p-mTOR (S2448) with a maximum decrease at 60′, whereas there was no significant change in the total mTOR levels under all experimental conditions (Fig. 1A and B) in comparison with either the control (no treatment) or the cells treated with the vehicle, DMSO. Rapamycin treatment resulted in an increase in the expression of total NMT1 levels over time, with a maximum increase of 6-fold at 360′ compared to control cells whereas, no significant change in NMT1 levels in cells treated with DMSO was observed (Fig. 1A and C). The total NMT1 expression was normalized against *β*-actin. Rapamycin treatment showed a correlation between the levels of p-mTOR (S2448)/total mTOR levels and total NMT1/*β*-actin levels, whereas, there was no significant change when the cells were treated with DMSO alone (Fig. 2). Four datasets were produced and were used for model calibration and selection.

### Modelling mTOR-NMT1 without rapamycin

**Figure 3:**
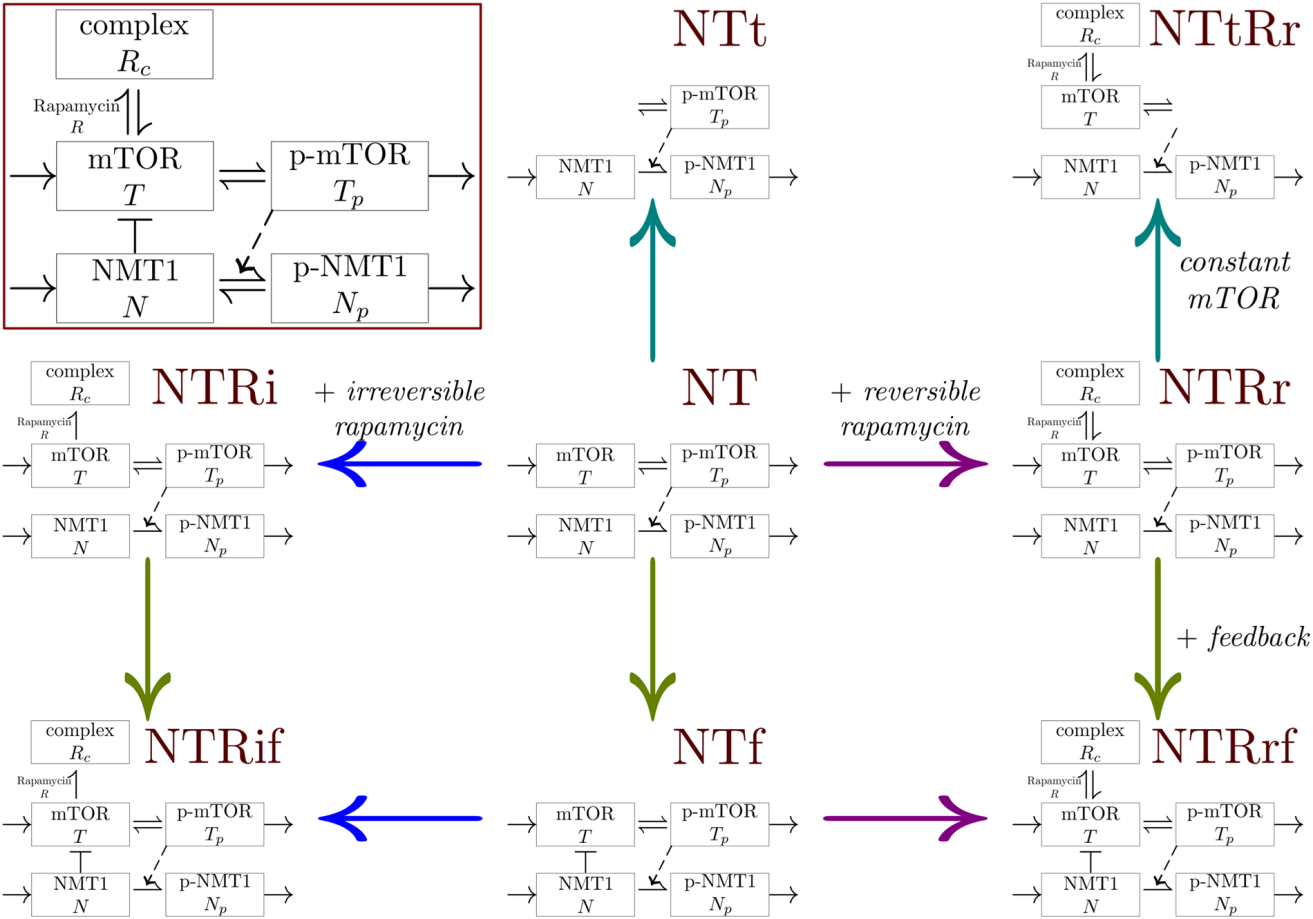
Derivation of the models studied, starting from model NT and applying multiple assumptions (constant total mTOR, feedback regulation of mTOR by NMT1 and rapamycin binding); corresponding graphs of interactions are in the background. For models with constant total mTOR, only the dynamics of *T* and *R*_*c*_ are considered with rapamycin, thus *T*_*p*_ = *T*_*t*_ − *T* − *R*_*c*_ while without rapamycin only *T*_*p*_ is explicitly described then *T* = *T*_*t*_ − *T*_*p*_. One may note that there is no model with constant total mTOR and irreversible rapamycin binding or feedback, to ensure biologically realistic dynamics. (Inset) Schematic relations between the model variables, corresponding to System (1). The dashed arrow indicates that p-mTOR is activating the phosphorylation of NMT1. The bar-headed line represents an inhibition and the arrows indicate a transfer or an activation.

**Figure 4:**
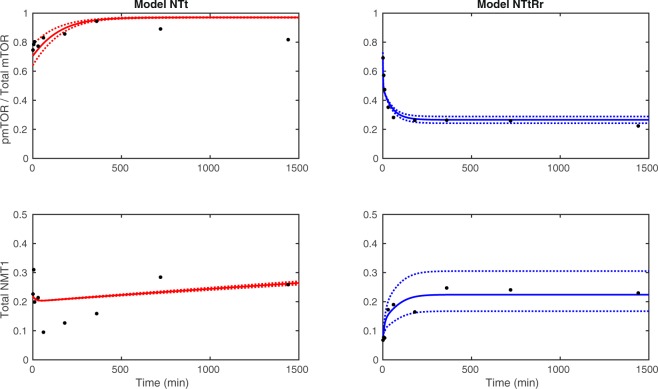
Best fit for models NTt (without rapamycin, on the left) and NTtRr (with rapamycin, on the right) for dataset 2 (solid line), with the envelope (dotted lines) corresponding to a variation of ±10% of the nominal parameter values (best set of parameters).

The three models, NT, NTt and NTf are considered to investigate the dynamics of the mTOR-NMT1 system without perturbations due to rapamycin treatment (see Fig. 3). Table 1 provides a summary of the characteristics and assumptions of the models; the core assumption of these models is the regulation of NMT1 phosphorylation by p-mTOR. All models have been studied analytically, as detailed in the Supplementary material. Using the four control datasets, parameter values for each model have been optimized to fit the experimental data. All these models are able to reproduce the trends in the proportion of phosphorylated mTOR and total NMT1 observed for all experimental datasets without rapamycin (see Figs 4 and S4). Experimental observations combined with the modelling approach validate the regulation of NMT1 by mTOR.

**Table 1:** Collection of models studied with the number of parameters *p*, assumptions on rapamycin binding, feedback regulation of mTOR by NMT1 and mTOR dynamics (s/d meaning an explicit synthesis and degradation of mTOR).

Model	*p*	rapamycin	feedback	mTOR
NT	10	no	no	s/d
NTt	9	no	no	constant
NTf	11	no	yes	s/d
NTRr	12	reversible	no	s/d
NTtRr	11	reversible	no	constant
NTRi	12	irreversible	no	s/d
NTRrf	13	reversible	yes	s/d
NTRif	13	irreversible	yes	s/d

Then the effects of explicit synthesis and degradation of mTOR components and inhibition of mTOR by NMT1 on the dynamics are investigated. For datasets 1 to 3, model NTt, characterized by a constant endogenous level of total mTOR (no explicit synthesis and degradation) and no feedback regulation of mTOR by NMT1, is selected as the best model with a strong level of evidence compared to models NT and NTf. Model NTt has the lowest AIC_*c*_ among the three models considered and its Akaike weight is over 0.9 for these datasets (see Table 2). Moreover, the evidence ratio for model NTt compared to the second best model NT is higher than 15 for each dataset. For dataset 4, model NTt is also selected as the best model, however with a lower Akaike weight of 0.6. Furthermore, a feedback regulation of mTOR by NMT1 is less likely to occur; the Akaike weight of the feedback assumption is lower than 0.01 for each dataset.

**Table 2:** Corrected Akaike Information criteria AICc_*i*_ and Akaike weights *w*_*i*_ for models with and without rapamycin for datasets 1 to 4, with *k*_*i*_ = *p* + 1 the number of parameters considered to compute the AICc_*i*_, with *i* denoting the model considered. The weights corresponding to each assumption are obtained by summing the weights of the models verifying the assumption (see Table 1).

Models		Dataset 1		Dataset 2		Dataset 3		Dataset 4	
	*k*_i_	AICc_*i*_	*w*_*i*_	AICc_*i*_	*w*_i_	AICc_*i*_	*w*_*i*_	AICc_*i*_	*w*_*i*_
(a) Without rapamycin									
NT	11	−6.9	0.062	−101.9	0.033	−67.2	0.045	−109.2	0.379
NTt	10	−12.4	**0**.**936**	−108.6	**0**.**967**	−73.5	**0**.**957**	−110.2	**0**.**609**
NTf	12	−0.7	0.003	−92.9	10^−4^	−61.2	0.002	−102.4	0.012
**Assumptions**									
Feedback			0.003		0.0004		0.002		0.01
No feedback			**0**.**997**		**0**.**9996**		**0**.**998**		**0**.**99**
(b) With rapamycin									
NTRr	13	−2.3	0.393	−107.1	10^−4^	−75.9	0.025	−58.8	0.407
NTtRr	12	−1.4	0.248	−124.9	**0**.**997**	−83.1	**0**.**929**	−58.2	0.305
NTRi	13	−2.0	0.351	−113.5	0.003	−76.7	0.039	−58.1	0.282
NTRrf	14	7.0	0.004	−91.6	10^−8^	−73.2	0.007	−48.0	0.002
NTRif	14	6.6	0.005	−102.4	10^−5^	−66.6	10^−4^	−49.4	0.004
**Assumptions**									
Reversible			**0**.**64**		**0**.**9967**		**0**.**961**		**0**.**714**
Irreversible			0.36		0.0033		0.039		0.286
Feedback			0.008		10^−5^		0.007		0.006
No feedback			**0**.**992**		**0**.**99999**		**0**.**993**		**0**.**994**

**Figure 5:**
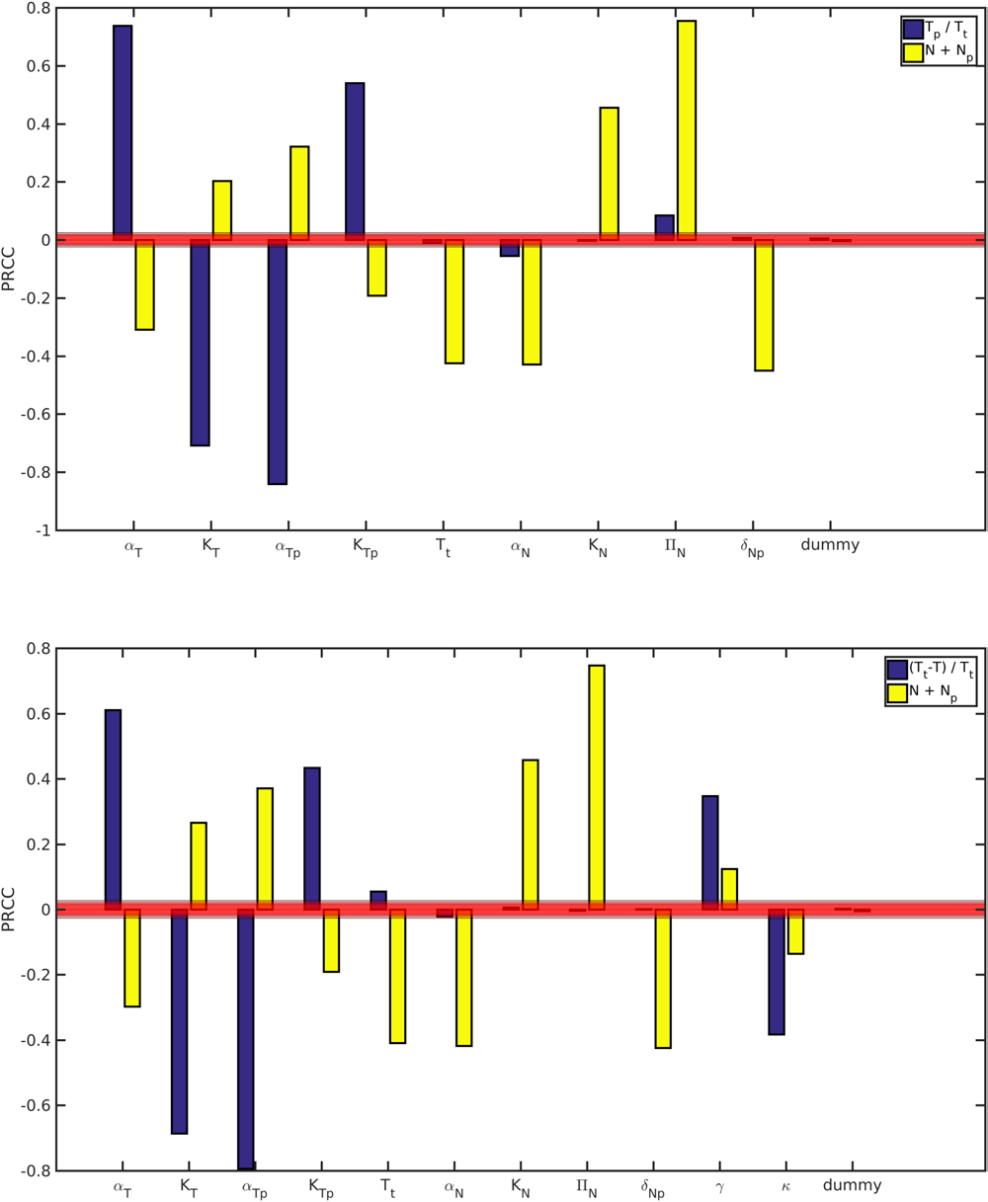
Global sensitivity analysis of the proportion of p-mTOR *T*_*p*_/*T*_*t*_ and total NMT1 *N* + *N*_*p*_, using LHS-PRCC for Models NTt (top) and NTtRr (bottom) at time *t* = 2880 min. The red area determines the area of significance (levels 0.05, 0.01 and 0.001).

In the following, the sensitivity and robustness of the best model NTt to parameters were investigated in details. The LHS-PRCC global sensitivity analysis of model NTt indicates that the proportion of p-mTOR and total NMT1 are significantly sensitive to at least one parameter (see Fig. 5) and that no parameter is negligible. One may note that total NMT1 is directly sensitive to all parameters, in particular parameters governing the dynamics of mTOR components.

Figure 4 displays trajectories of the model NTt with dataset 2; the dotted lines indicate the minimum and maximum trajectories corresponding to a ±10% variation of the nominal parameter values obtained for dataset 2. Model responses for the proportion of p-mTOR and total NMT1 appear to be robust to small changes in parameter values. The estimated parameter values are of the same order of magnitude for all 4 datasets, with some small differences resulting from variations in the experimental levels of NMT1 and p-mTOR (see Figure S6).

### Modelling mTOR-NMT1 with rapamycin

The dynamics of mTOR and NMT1 is now studied with rapamycin treatment by considering five models, NTRr, NTtRr, NTRi, NTRrf and NTRif (see Fig. 3). Table 1 provides a summary of the assumptions of these models. These five models are calibrated using four datasets treated with rapamycin. All models are able to reproduce the trends observed in experimental data with rapamycin, in particular a decrease in the proportion of p-mTOR and an increase in total NMT1 (see Figs 4 and S5). Similarly to the control, model responses support the regulation of NMT1 by p-mTOR in the presence of rapamycin.

Considering these five models allowed us to test the assumptions previously investigated in the control case, as well as the reversibility of rapamycin effect (see Fig. 3 and Table 1).

For datasets 2 and 3, model NTtRr, characterized by a reversible effect of rapamycin, a constant total mTOR and an absence of feedback, is the most likely to be the best model; it has the lowest AIC_*c*_ and a weight above 0.9 (see Table 2 and Fig. 4). The best models with and without rapamycin are based on the same assumptions. Moreover, the conclusions of the global sensitivity analysis of model NTtRr are the same as for model NTt (see Fig. 5); parameters common to both models have similar impacts.

However, for datasets 1 and 4, multiple models have similar AIC_*c*_ and Akaike weights, not allowing us to determine the best model with a sufficient support. For instance, the best model selected for datasets 2 and 3, NTtRr, overestimates the initial value of p-mTOR on dataset 1, leading to a higher error (see Supplementary material). Even if the best model cannot be selected for datasets 1 and 4, a reversible effect of rapamycin and an absence of feedback are the most probable assumptions, similarly to the other datasets (see Table 2).

Estimated parameter values are of the same order of magnitude for all four datasets for model NTtRr (see Figure S6); parameters common to models NTt and NTtRr have similar values.

## Discussion

In our earlier studies using *in-vitro* kinase assay, we have demonstrated that Akt phosphorylates NMT1, which causes a decrease in its activity^29^. Since mTORC1 is a downstream target of Akt we were interested in determining the effect of mTOR inhibition on NMT1. Rapamycin binds to the intracellular receptor, FKBP12. The FKBP12-rapamycin complex binds to mTOR and inhibits its autophosphorylation at S2448^39^. Rapamycin has also been proposed to inhibit mTOR by destabilizing the mTOR-raptor complex^40^. The regulation of NMT1 by mTOR was previously not known. Upon inhibiting mTOR with rapamycin we observed an increased expression of NMT1 in a time dependent manner whereas, DMSO, the vehicle failed to show any significant changes in the total NMT1 levels (see Fig. 1).

Furthermore, we performed similar experiments in another ER positive T47D breast cancer cell line. The ER positive T47D breast cancer cells were treated with rapamycin for the same concentration and time points as that of MCF7 cells. The expression pattern of NMT1 upon rapamycin treatment in T47D was found to be similar to that of MCF7 cells (see Figure S3). The dynamic expression of p-mTOR and NMT1 when treated with DMSO (vehicle control) or rapamycin in T47D was similar to that of MCF7 cells. This data confirms that the regulation of NMT1 by mTOR is not a cell line dependent phenomenon. We used the best model obtained from MCF7 data fitting, NTt and NTtRr, to fit the data obtained by treating T47D cells with or without rapamycin. Our results indicate that the model outputs are a good representation of experimentally obtained data (see Figures S9 to S11). Moreover, estimates of parameter values for both the cell lines, MCF7 and T47D are in similar ranges.

It is possible that activated mTOR acts on NMT1 and primes it for a proteasome-mediated degradation. Ubiquitin-mediated proteasome degradation of proteins is involved during stress-response, signal transduction, antigen processing, DNA-repair, transcriptional regulation, apoptosis, lytic degradation of transcription factors, misfolded proteins, parasitic proteins, etc^41–46^. Zhao *et al*. reported that inhibition of mTORC1 by rapamycin for as long as 16 h caused a reduction in proteolysis due to decreased proteasome expression^47^. The study by Zhang *et al*. supports our observation that the inhibition of mTOR by rapamycin could possibly prevent proteasome-mediated degradation of NMT1^48^.

There are a number of proteins, which are degraded due to phosphorylation that makes them susceptible to ubiquitination and proteasome-mediated degradation such as glycogen synthase kinase-3 (GSK-3), which phosphorylates *β*-catenin thus tagging it with ubiquitin for proteasome mediated degradation. Many cell cycle regulated proteins such as cyclins are subjected to proteasomal-mediated degradation for the transition from one phase of cell cycle to another.

Studies in yeast clearly demonstrate the role of NMT in the cell morphogenesis through the regulation of proteasomal activity. A temperature sensitive mutant swoF1 with mutation in NMT encoding gene in *Aspergillus nidulans* could not maintain growth during cell elongation. It was further demonstrated that swoF1 NMT mutants had an increased 26S proteosomal activity^49^. In fact, the defect of NMT gene in swoF1 mutants could be partially reversed by the introduction of a second mutation, which mutated 20S proteasomal subunit. All these studies strongly support the loss of NMT activity in mutant fungus is associated with an increased proteasome activity. Research led by Khandelwal have demonstrated that Akt, a protein upstream of mTOR localizes into the nucleus, where it could act as a transcription factor, so the other alternative could be that as a transcription factor Akt could be a down regulator of transcription of NMT1 gene, thus blocking the expression of NMT1 mRNA. Although, the phosphorylation of NMT1 by Akt has been demonstrated. It is unknown whether Akt regulates the transcription of NMT1 in the nucleus^50^.

Considering the collection of mathematical models and data, we can validate the assumption of a regulation of NMT1 phosphorylation downstream of mTOR. Under control and perturbed conditions, a probable scenario for the mTOR-NMT1 regulation is characterized by a constant endogenous level of total mTOR and an absence of feedback regulation of mTOR by NMT1. Moreover, rapamycin appears to have a reversible effect on mTOR. The assumption of a constant endogenous level of total mTOR, shared by the best models with and without rapamycin, is in agreement with previously published models of mTOR pathways^32–34^. These previous models described explicitly signalling upstream of mTOR by using mass-action law. As our models are driven and depend on experimental data for their calibration, only a phenomenological representation of mTOR upstream regulation (described as its phosphorylation) is used in the current work. Despite this phenomenological representation of the upstream regulation of mTOR some of our estimates (maximal rates of phosphorylation and dephosphorylation of mTOR) can be compared to those from previously published work and are of the same order of magnitude after rescaling^32^. However, to the best of our knowledge, as this work introduces the first models coupling the dynamics of both mTOR and NMT1, estimates for other parameters are not presently available in the literature for comparison.

When considering control experimental data and the corresponding three models, the model selection is conclusive and select the same best model. However, when including the effect of rapamycin, several models are similarly good for some datasets and it is thus difficult to select one of these models as the best. In the models, we assumed that rapamycin concentration in the cell remained constant over time and that rapamycin only interacts with mTOR. As rapamycin was used to perturb mTOR and to keep the models simple, the interaction between mTOR and rapamycin was modelled as reversible or irreversible using mass action type terms. However, it is possible that rapamycin dynamics is more complex than actually modelled with respect to its action on mTOR and the consequent effect on NMT1. Furthermore, rapamycin could induce further intracellular changes because of slight variations in the expression data due to differences in passages of MCF7 cells used for the trials, despite this the biological trend remained consistent in four trials. The modelling work reported in this study is the first step in the construction of a general model of this cellular pathway for future studies, which can aide in determining regulations upstream of mTOR and downstream of NMT1.

## Methods

### Cell culture and treatment

Human breast adenomacarcinoma, MCF7 cells were a kind gift from Dr. Leigh Murphy (University of Manitoba, Canada). The cells were maintained in Dubecco’s modified Eagle medium (DMEM) supplemented with 10% fetal bovine serum (FBS), 2 mM L-glutamine, glucose, 100 U/ml of penicillin and 100 *μ*g/ml streptomycin at 37 °C with 5% CO2 in a humidified incubator. MCF7 cells were grown in 100 mm tissue culture dishes.

### Western Blot Analyses

MCF7 cells of ~75–85% confluence were starved in the culture medium depleted of FBS for 6 h and treated with either 100 nM rapamycin or the vehicle, DMSO for a period of 5′, 10′, 30′, 60′, 180′, 360′, 720′ and 1440′. At indicated times the cells were washed with cold phosphate buffered saline (PBS). Following the treatment total protein was extracted by lysing the cells in a lysis buffer containing HEPES (pH 7.4, 50 mM), sucrose (150 mM), sodium orthovanadate (2 mM), *β*-glycerophosphate (80 mM), sodium fluoride (10 mM), sodium pyrophosphate (10 mM), sodium EGTA (2 mM), sodium EDTA (2 mM), triton X-100 (1%), SDS (0.1%), phenyl methyl sulphonyl fluoride (PMSF; 1 mM) and protease inhibitor cocktail (1%; Sigma Aldrich). The cells were scraped, collected in an eppendorf and allowed to stand on ice for 10 min. The lysates were spun at 13,000 rpm for 10′ at 4 °C, the pellet was discarded and the supernatant was collected and stored at −20 °C for future use. The protein lysates (30 *μ*g) were resolved in 8% and 10% SDS-PAGE, transferred onto polyvinylidene difluoride (PVDF) membranes (Amersham Biosciences, Quebec, Canada) and blocked with 5% milk dissolved in PBS with tween-20 (PBST) for 1 hour at room temperature. The membranes were then probed with rabbit polyclonal antibody against p-mTOR S2448 (Sigma, Canada) and mouse monoclonal anti-NMT1 antibody (a kind gift from VastCon Inc, Canada). The excess antibodies were removed by washing the membranes three times with PBST, 10′ each and incubated with HRP-conjugated secondary antibody for an hour at room temperature. The membranes were washed three times in PBST, 10′ each and visualization was done using ClarityWestern ECL substrate (Bio-Rad) reagent and a Molecular Imager ChemiDoc XRS System (Bio-Rad) and Image Lab software Version 3.0. The membranes were stripped and re-probed with antibodies against total mTOR and *β*-actin. *β*-actin is a housekeeping protein and was used as a loading control. Densitometric analysis was performed using Image lab software version 3.0 and relative band intensities were presented as a ratio of phosphorylated mTOR (S2448) over total mTOR or NMT1 over *β*-actin, compared with control (no treatment, 0′). Four independant experiments were performed and the representative blots are shown in Figs 1 and S1.

### Mathematical models

We designed a collection of eight models describing the dynamics of mTOR and NMT1. These eight models result from alternative hypotheses, which are difficult to test experimentally,

- Does the regulation of endogenous levels of mTOR components impact the dynamics?
- Does NMT1 have a negative feedback effect on mTOR?
- Is the effect of rapamycin on mTOR reversible or irreversible?

The models are based on biological assumptions and designed to accommodate the available data: intracellular p-mTOR, total mTOR and total NMT1 over time, with or without perturbations due to rapamycin treatment. The upstream regulation of mTOR is not explicitly included in the models but phenomenologically described in the phosphorylation of mTOR. The core of all these models is the regulation of NMT1 phosphorylation by p-mTOR.

Hence, we consider the dynamics of mTOR *T*, p-mTOR *T*_*p*_, NMT1 *N*, p-NMT1 *N*_*p*_ and the complex rapamycin-mTOR *R*_*c*_, as follows,

1 $$\begin{array}{rcl}\frac{{\rm{d}}T}{{\rm{d}}t} & = & \mathop{\overbrace{\frac{-{\alpha }_{T}T}{{K}_{T}+T}}}\limits^{{\rm{phosphorylation}}}+\mathop{\overbrace{\frac{{\alpha }_{{T}_{p}}{T}_{p}}{{K}_{{T}_{p}}+{T}_{p}}}}\limits^{{\rm{dephosphorylation}}}+\mathop{\overbrace{{{\rm{\Pi }}}_{T}}}\limits^{{\rm{synthesis}}}+\mathop{\overbrace{f(T,N)}}\limits^{{\rm{feedback}}}+\mathop{\overbrace{g(T,{R}_{c}),}}\limits^{{\rm{rapamycin}}\,{\rm{effect}}}\\ \frac{{\rm{d}}{T}_{p}}{{\rm{d}}t} & = & \mathop{\overbrace{\frac{{\alpha }_{T}T}{{K}_{T}+T}}}\limits^{{\rm{phosphorylation}}}-\mathop{\overbrace{\frac{{\alpha }_{{T}_{p}}{T}_{p}}{{K}_{{T}_{p}}+{T}_{p}}}}\limits^{{\rm{dephosphorylation}}}-\mathop{\overbrace{{\delta }_{{T}_{p}}{T}_{p},}}\limits^{{\rm{degradation}}}\\ \frac{{\rm{d}}N}{{\rm{d}}t} & = & \mathop{\overbrace{-\frac{{\alpha }_{N}{T}_{p}N}{{K}_{N}+N}}}\limits^{{\rm{phosphorylation}}}+\mathop{\overbrace{{{\rm{\Pi }}}_{N},}}\limits^{{\rm{synthesis}}}\\ \frac{{\rm{d}}{N}_{p}}{{\rm{d}}t} & = & \mathop{\overbrace{\frac{{\alpha }_{N}{T}_{p}N}{{K}_{N}+N}}}\limits^{{\rm{phosphorylation}}}-\mathop{\overbrace{{\delta }_{{N}_{p}}{N}_{p},}}\limits^{{\rm{degradation}}}\\ \frac{{\rm{d}}{R}_{c}}{{\rm{d}}t} & = & \mathop{\overbrace{h(T,{R}_{c}\mathrm{).}}}\limits^{{\rm{rapamycin}}\,{\rm{effect}}}\end{array}$$

Figure 3 displays a schematic representation of the interactions between the variables corresponding to the system defined in (1), as well as a graphical representation of the relationship between models in the collection. Model assumptions are summarized in Table 1.

The regulation of the endogenous level of mTOR is modelled by the synthesis of mTOR and the degradation of p-mTOR. When the synthesis of mTOR and the degradation of p-mTOR are not explicitly described in the model, the total amount of mTOR *T*_*t*_ is assumed to be constant over time and the corresponding models are labelled constant total mTOR. We do not explicitly model the concentration of rapamycin *R* in the cell, assuming it is abundant and available throughout the experiment, and consider that it remains constant for the timescale considered. The absorption of rapamycin into the cell is not the focus of the study and is thus not described. When needed, enzymatic reactions are assumed to follow Michaelis-Menten dynamics^51,52^. To reduce the number of parameters and the complexity of (1) and without experimental data to quantify p-NMT1, the dephosphorylation of p-NMT1 is not considered; however, we proved mathematically that it does not change the dynamics of the models. For all models there exists at least a positive stable equilibrium under some conditions on parameters. Mathematical analyses of all models are detailed in Supplementary material, see S2.1.

#### Modelling mTOR-NMT1 without rapamycin

Models NT, NTt and NTf consider only mTOR and NMT1 without rapamycin (see Table 1 and Fig. 3), this implies that *g*(*T*, *R*_*c*_) = *h*(*T*, *R*_*c*_) = 0. Model NT describes the dynamics of mTOR, p-mTOR, NMT1 and p-NMT1 with synthesis and degradation of mTOR components and without feedback *f*(*T*, *N*) = 0. Model NTt is derived from model NT by setting a constant total mTOR *T*_*t*_ = *T* + *T*_*p*_, which allowed us to reduce the number of equations and describe only the dynamics of p-mTOR *T*_*p*_. Model NTf allowed us to test the assumption of a feedback regulation of mTOR by NMT1 using *f*(*T*, *N*) = −*βNT* (Table 1 and Fig. 3). We did not consider a negative feedback and constant total mTOR at the same time, as the resulting dynamics is biologically unrealistic (no positive steady state). Model equations are provided in Supplementary material, see S2.1.

#### Modelling the effect of rapamycin

Models NTRr, NTtRr, NTRi, NTRrf and NTRif, additionally describe the interaction between mTOR and rapamycin (see Table 1 and Fig. 3). In models with an irreversible binding of rapamycin, *g*(*T*, *R*_*c*_) = −*γRT* and $h(T,{R}_{c})=\gamma RT-{\delta }_{{R}_{c}}{R}_{c}$. A reversible binding is described by *g*(*T*, *R*_*c*_) = −*γRT* + *κR*_*c*_ and *h*(*T*, *R*_*c*_) = *γRT* − *κR*_*c*_.

From model NT are derived two models by adding a reversible (NTRr) or irreversible (NTRi) binding to rapamycin. Models NTRrf and NTRif were obtained by adding the effect of rapamycin to model NTf or, alternatively, by adding the description of the feedback to models NTRr or NTRi (see Fig. 3). Finally, model NTtRr was derived from model NTRr and characterized by a constant total mTOR, when the rapamycin binding is reversible. In this case, *T*_*t*_ = *T* + *T*_*p*_ + *R*_*c*_ and we consider only the dynamics of mTOR *T* and the complex rapamycin-mTOR *R*_*c*_ (p-mTOR *T*_*p*_ is thus deduced from *T*_*t*_, *T* and *R*_*c*_). Similarly to the control, the constant total mTOR assumption is not considered with irreversible binding and/or feedback. Model equations are provided in Supplementary material, see S2.1.

#### Model calibration and selection

In order to calibre the outputs of the models to the experimental data, we first estimated parameter values for each model; the number of estimated parameters for each model is listed in Table 1. To reduce the number of estimated parameters, we set *R* = 1, as we assumed that the concentration of rapamycin inside the cell remains constant during the course of the experiment. For each of the four datasets, the following experimental data are measured at *m* = 9 time points: total mTOR ${T}_{total}^{exp}$, p-mTOR ${T}_{p}^{exp}$ and total NMT1 ${N}_{total}^{exp}$, all normalized by *β*-actin at the time point considered. For the four datasets, the residual sum of squares between experimental and simulated data for model *i* is defined as follows

2 $${{\rm{RSS}}}_{i}=\sum _{j=1}^{m}\,[{({T}_{p}^{exp}({t}_{j})-{T}_{p}^{i}({t}_{j}))}^{2}+{({T}_{total}^{exp}({t}_{j})-{T}_{total}^{i}({t}_{j}))}^{2}+{({N}_{total}^{exp}({t}_{j})-{N}_{total}^{i}({t}_{j}))}^{2}],$$

where *t*_*j*_ are the *m* time points, ${T}_{total}^{i}({t}_{j})={T}^{i}({t}_{j})+{T}_{p}^{i}({t}_{j})$ and ${N}_{total}^{i}({t}_{j})={N}^{i}({t}_{j})+{N}_{p}^{i}({t}_{j})$ account for the responses in model *i*. To obtain the best fit and thus the best set of parameters, we minimized the residual sum of squares between the experimental data and the model output with a genetic algorithm, and we repeated this procedure multiple times (all the fitting procedure was performed using Matlab R2016b). For a given dataset, a model will thus be associated with a nominal set of parameters.

Then, model selection was used to identify the best model and characterize the required mechanisms. In order to select the best model, given the collection of models and experimental data, we computed the Akaike Information Criterion (AIC_*i*_) for each model *i*, which takes into account the number of estimated parameters and the goodness of the fit^53,54^ (see Table 2), as follows:

3 $${{\rm{AIC}}}_{i}=n\,\mathrm{ln}\,(\frac{{{\rm{RSS}}}_{i}}{n})+2{k}_{i},$$

where *n* = 3*m* is the number of data points used for parameters estimation and *k*_*i*_ is the number of estimated parameters for model *i*, including the estimation of RSS_*i*_/*n*. As the number of data points was small (*n* = 27 for each dataset) in comparison to the number of parameters we used the AIC corrected for small sample sizes instead of the AIC, as follows:

4 $${{\rm{AICc}}}_{i}={{\rm{AIC}}}_{i}+\frac{2{k}_{i}({k}_{i}+\mathrm{1)}}{n-{k}_{i}-1}=n\,\mathrm{ln}\,(\frac{{{\rm{RSS}}}_{i}}{n})+2{k}_{i}\frac{n}{n-{k}_{i}-1}\mathrm{.}$$

Recall the best model is the model with the lowest AICc^53^.

For the collection of models, we can then calculate the Akaike weight *w*_*i*_, which can be interpreted as the probability that the model *i* is the best model given the experimental data and the set of models considered:

5 $${w}_{i}=\frac{\exp (\,-\,{{\rm{\Delta }}}_{i}\mathrm{/2)}}{{\sum }_{j=1}^{N}\,\exp (\,-\,{{\rm{\Delta }}}_{j}\mathrm{/2)}},$$

with *N* the total number of models in the collection and ${{\rm{\Delta }}}_{i}={{\rm{AICc}}}_{i}-\mathop{{\rm{\min }}}\limits_{j=\mathrm{1..}N}\,({{\rm{AICc}}}_{j})$.

To quantify the probability of each assumption, we computed their respective weights by summing the Akaike weights of the models verifying the assumption. Thus, the weight of models with reversible (resp. irreversible) rapamycin effect will be obtained by summing the weights of models including this assumption namely NTRr, NTtRr and NTRrf (resp. NTRi and NTRif). The weight of the feedback assumption corresponds to the weight of model NTf without rapamycin or models NTRrf and NTRif with rapamycin.

#### Robustness and sensitivity analysis

For the best models, we evaluated the global sensitivity to parameters, using the LHS-PRCC (Partial Rank Correlation Coefficient with Latin Hypercube Sampling) method^55,56^ (see Fig. 5). Sets of parameter values were sampled uniformly using LHS (the sampling interval includes nominal parameter values). We then eliminated the parameters not satisfying the existence condition for a positive equilibrium (see Supplementary materials Section S2.1). These constraints on parameter values were imposed to ensure a biologically relevant behavior for the models. Then models were simulated until time 2880 minutes (twice the experimental time) to ensure equilibrium is reached. The proportion of p-mTOR and total NMT1 at steady state were used to compute the PRCC (see Fig. 5). The sensitivities of all non aggregated variables are shown in Figure S13.

In order to determine the impact of small variations of parameters on the best model outputs, we sampled random sets of parameters, with each parameter following a uniform distribution between 0.9 and 1.1 times the nominal value obtained by fitting. This allowed us to study the effect of parameter fluctuations around the nominal value, assess the robustness of models and define minimal and maximal values of the outputs over time (see Fig. 4 and Supplementary materials Section S2.2.2).

## Electronic supplementary material

Supplementary information
