# Supplementary material for: Investigation of Novel Regulation of N-myristoyltransferase by Mammalian Target of Rapamycin in Breast Cancer Cells
Source: Sci Rep. 2018 Aug 28;8:12969. doi: 10.1038/s41598-018-30447-0 (PMC6113272; doi:10.1038/s41598-018-30447-0)
Supplement: Supplementary file 1 — Supplementary information [file 41598_2018_30447_MOESM1_ESM.pdf]

Investigation of Novel Regulation of N-myristoyltransferase by Mammalian Target of Rapamycin in Breast Cancer cells Supplementary Information

Marine Jacquier, Shiby Kuriakose, Apurva Bhardwaj, Yang Zhang, Anuraag Shrivastav, Stéphanie Portet and Shailly Varma Shrivastav

S1 Western blot analyses

S1.1 MCF7 cell line

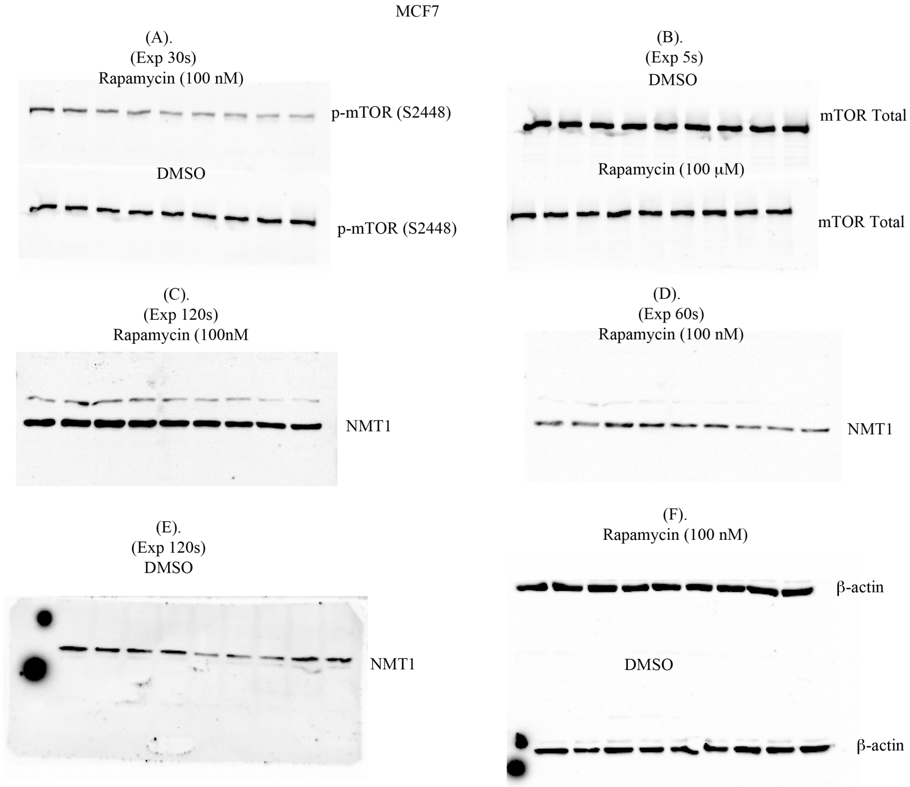

Figure S1: Western blot analyses of MCF7 cells starved for 6 h in serum-deprived media followed by treatment with either rapamycin (100 nM) or DMSO (vehicle) for various time points and protein lysates were made. Volume corresponding to 30 μg of proteins was separated on SDS-PAGE and Western blot analyses were carried out as described in the Materials and Methods section. Western blot images in the figure represent lane 1 is 0', Lane 2 is 5', lane 3 is 10', lane 4 is 30', lane 5 is 60', lane 6 is 180', lane 7 is 360', lane 8 is 720' and lane 9 is 1440'. Rapamycin and DMSO treated cell lysates were probed with (A) p-mTOR (S2448) and both the membranes were imaged together. The membranes were then stripped and re-probed with (B) total mTOR, which were also imaged together. Western blot analyses of rapamycin treated lysates were probed with NMT1 (C,D and E). The membranes were then stripped and re-probed with (F) β-actin which was also imaged together. Exp = exposure time.

S1.2 Additional cell line: T47D

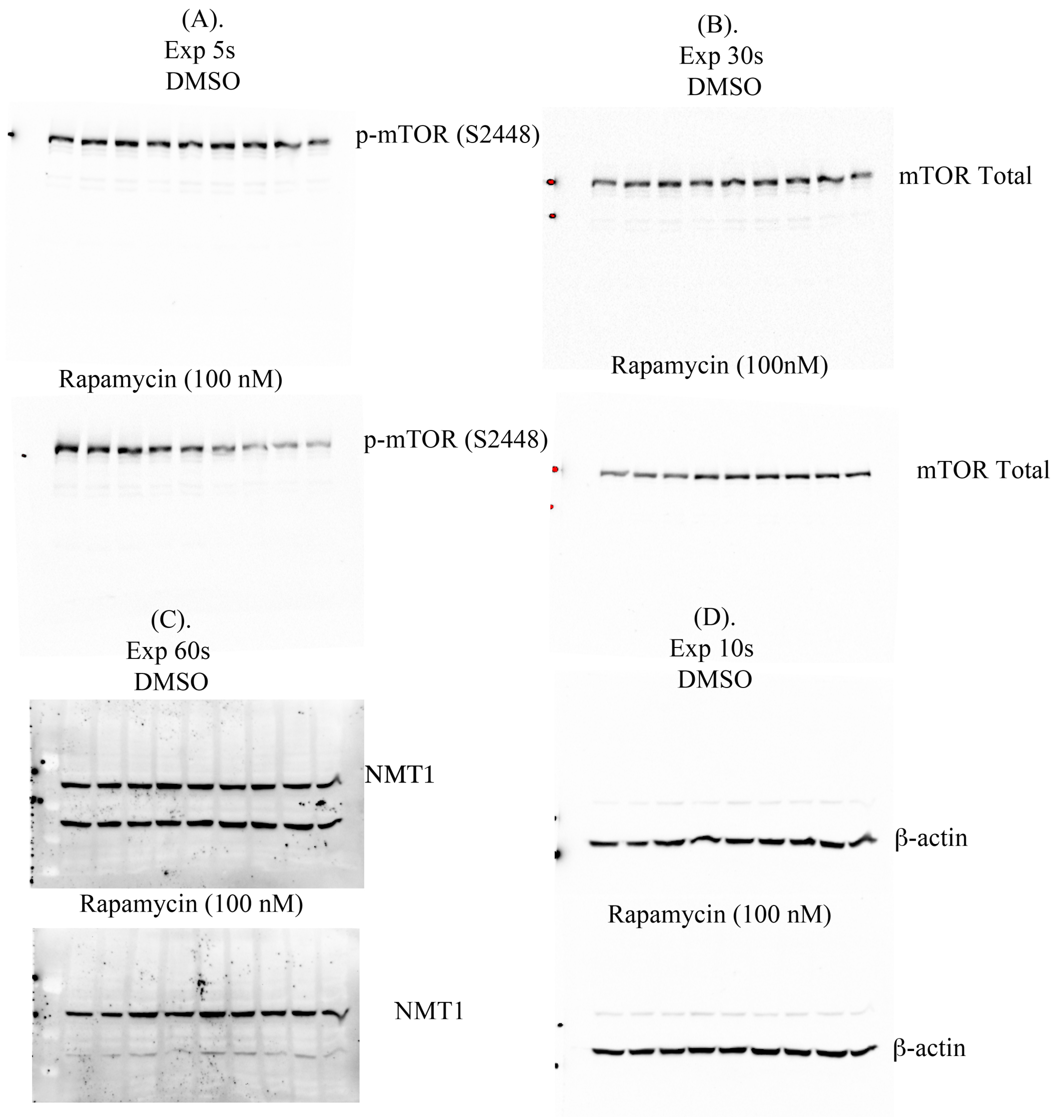

Figure S2: Western blot analyses of T47D cells starved for 6 h in serum-deprived media followed by treatment with either rapamycin (100 nM) or DMSO (vehicle) for various time points and protein lysates were made. Volume corresponding to 30 μg of proteins was separated on SDS-PAGE and Western blot analyses were carried out as described in the Materials and Methods section. Western blot images in the figure represent lane 1 is 0', Lane 2 is 5', lane 3 is 10', lane 4 is 30', lane 5 is 60', lane 6 is 180', lane 7 is 360', lane 8 is 720' and lane 9 is 1440'. Rapamycin and DMSO treated cell lysates were probed with (A) p-mTOR (S2448). The membranes were then stripped and re-probed with (B) total mTOR. Western blot analyses of rapamycin or DMSO treated lysates were probed with NMT1 (C). The membranes were then stripped and re-probed with (D) β-actin. Exp = exposure time.

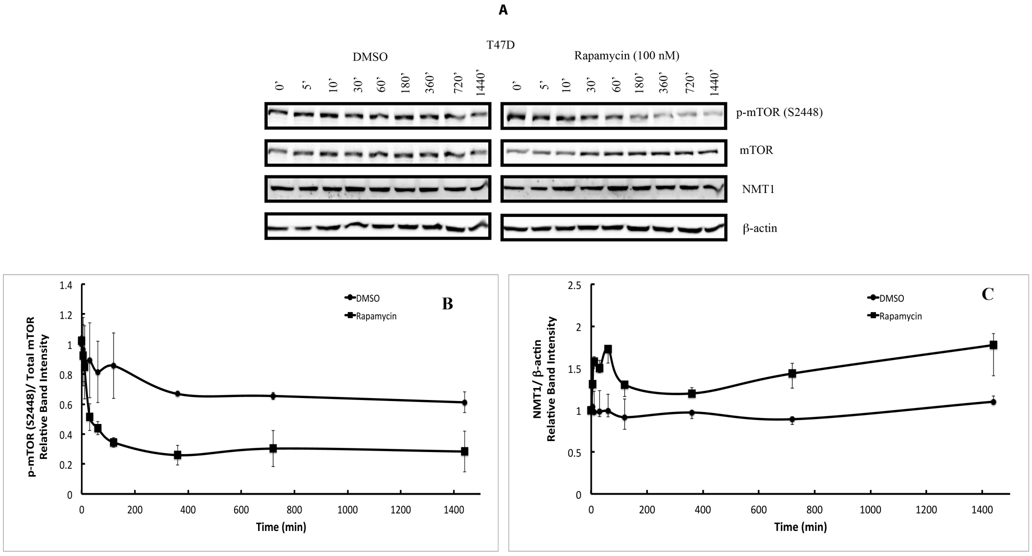

Figure S3: Rapamycin treatment decreased the phosphorylation of mTOR (S2448) and augmented NMT1 levels in a time-dependent manner. T47D cells at 70-80% confluence were starved and treated with rapamycin (100 nM) or DMSO for indicated time points and protein lysates were prepared as mentioned in the materials and methods section. (A). A representative Western blot analysis displaying the expression levels of p-mTOR (S2448), mTOR total, NMT1 and β-actin. The depiction of relative band intensities of p-mTOR (S2448)/total mTOR (B) and NMT1/β-actin (C) when T47D cells were treated with rapamycin or DMSO for indicated time points. All the experimental data were normalized against control 0' (no treatment) which is 1-fold. The data point is an average of two independent experiments and the bars represent the standard deviation between them. (T47D cells were cultured under the identical conditions as those of MCF7 cells as described in "Methods" section.)

S2 Mathematical models

S2.1 Mathematical results

We studied the dynamics of mTOR T, p-mTOR T_p, NMT1 N, p-NMT1 N_p and the complex rapamycin-mTOR R_c as described in System (1).

First, it can be shown that the positive orthant $\mathbb{R}^{5+}$ is invariant under the flow of Eq. (1), so we have the existence, uniqueness and nonnegativity of solutions. Those hold for all the models studied.

Here, we outline the mathematical results obtained from the qualitative analysis of the asymptotic behavior of the eight models considered in this work.

S2.1.1 NMT1 and mTOR dynamics without rapamycin

We describe the dynamics of mTOR and NMT1, without considering the dephosphorylation of p-NMT1. The following models will be considered with and without a feedback regulation of mTOR by NMT1.

S2.1.1.1 Without feedback – Models NT and NTt In the absence of feedback from NMT1 to mTOR, we consider two alternative models: one with explicit synthesis of mTOR and degradation of p-mTOR and one assuming a constant total mTOR $T_t = T + T_p$.

S2.1.1.1.1 Synthesis/degradation of mTOR components – Model NT Model NT is defined as

$$\frac{\mathrm{d}T}{\mathrm{d}t} = -\frac{\alpha_T T}{K_T + T} + \frac{\alpha_{T_p} T_p}{K_{T_p} + T_p} + \Pi_T, \tag{S1a}$$

$$\frac{\mathrm{d}T_p}{\mathrm{d}t} = \frac{\alpha_T T}{K_T + T} - \frac{\alpha_{T_p} T_p}{K_{T_p} + T_p} - \delta_{T_p} T_p, \tag{S1b}$$

$$\frac{\mathrm{d}N}{\mathrm{d}t} = -\frac{\alpha_N T_p N}{K_N + N} + \Pi_N, \tag{S1c}$$

$$\frac{\mathrm{d}N_p}{\mathrm{d}t} = \frac{\alpha_N T_p N}{K_N + N} - \delta_{N_p} N_p. \tag{S1d}$$

It can be shown that System (S1) has a unique positive equilibrium:

$$\begin{aligned}
T^* &= \frac{\Pi_T K_T}{\alpha_T - \Pi_T \left(1 + \frac{\alpha_{T_p}}{K_{T_p}\delta_{T_p} + \Pi_T}\right)} \left(1 + \frac{\alpha_{T_p}}{K_{T_p}\delta_{T_p} + \Pi_T}\right), \\
T_p^* &= \frac{\Pi_T}{\delta_{T_p}}, \\
N^* &= \frac{\Pi_N K_N \delta_{T_p}}{\Pi_T \alpha_N - \Pi_N \delta_{T_p}}, \\
N_p^* &= \frac{\Pi_N}{\delta_{N_p}},
\end{aligned} \tag{S2}$$

under the following existence condition

$$\frac{\Pi_N}{\alpha_N} < \frac{\Pi_T}{\delta_{T_p}} < \frac{(\alpha_T - \alpha_{T_p} - K_{T_p}\delta_{T_p}) + \sqrt{(\alpha_T - \alpha_{T_p} - K_{T_p}\delta_{T_p})^2 + 4\alpha_T K_{T_p}\delta_{T_p}}}{2\delta_{T_p}} \tag{S3}$$

Using linearization, we can show that the unique equilibrium defined in (S2) is locally asymptotically stable when it exists.

S2.1.1.1.2 Constant total mTOR T_t – Model NTt We assume that the total mTOR concentration T_t is constant, then $T_t = T_p + T$ and we reduce the dimension of System (S1) and obtain the model NTt.

$$\frac{\mathrm{d}T_p}{\mathrm{d}t} = \frac{\alpha_T(T_t - T_p)}{K_T + T_t - T_p} - \frac{\alpha_{T_p} T_p}{K_{T_p} + T_p}, \tag{S4a}$$

$$\frac{\mathrm{d}N}{\mathrm{d}t} = -\frac{\alpha_N T_p N}{K_N + N} + \Pi_N, \tag{S4b}$$

$$\frac{\mathrm{d}N_p}{\mathrm{d}t} = \frac{\alpha_N T_p N}{K_N + N} - \delta_{N_p} N_p. \tag{S4c}$$

System (S4) has a unique positive equilibrium (T_p^*, N^*, N_p^*) that exists when $\frac{\Pi_N}{\alpha_N} < T_p^* < T_t$ where

$$T_p^* = \begin{cases} \dfrac{\alpha_T K_{T_p} + \alpha_{T_p} K_T + (\alpha_{T_p} - \alpha_T)T_t - \sqrt{\Delta}}{2(\alpha_{T_p} - \alpha_T)} & \text{if } \alpha_T \neq \alpha_{T_p} \\ \dfrac{K_{T_p} T_t}{K_{T_p} + K_T} & \text{if } \alpha_T = \alpha_{T_p} \end{cases} \tag{S5}$$

with $\Delta = \left[\left(\alpha_T T_t - \alpha_{T_p} K_T\right) - \left(\alpha_{T_p} T_t - \alpha_T K_{T_p}\right)\right]^2 + 4\alpha_T \alpha_{T_p} K_T K_{T_p}$. The other components of the positive equilibrium are

$$\begin{aligned} N^* &= \frac{\Pi K_N}{\alpha_N T_p^* - \Pi_N}, \\ N_p^* &= \frac{\Pi_N}{\delta_{N_p}}. \end{aligned} \tag{S6}$$

One may note that the condition $\frac{\Pi_N}{\alpha_N} < T_p^*$ matches the lower bound condition (S3) for model NT. Using the linearization of System (S4), the equilibrium is locally asymptotically stable when it exists.

S2.1.1.2 With feedback – Model NTf We now assume a feedback regulation of mTOR by NMT1, mTOR is thus degraded proportionally to a function of NMT1 $f(N, T) = -\beta N T$ in model NTf

$$\frac{\mathrm{d}T}{\mathrm{d}t} = -\frac{\alpha_T T}{K_T + T} + \frac{\alpha_{T_p} T_p}{K_{T_p} + T_p} + \Pi_T - \beta N T, \tag{S7a}$$

$$\frac{\mathrm{d}T_p}{\mathrm{d}t} = \frac{\alpha_T T}{K_T + T} - \frac{\alpha_{T_p} T_p}{K_{T_p} + T_p} - \delta_{T_p} T_p, \tag{S7b}$$

$$\frac{\mathrm{d}N}{\mathrm{d}t} = -\frac{\alpha_N T_p N}{K_N + N} + \Pi_N, \tag{S7c}$$

$$\frac{\mathrm{d}N_p}{\mathrm{d}t} = \frac{\alpha_N T_p N}{K_N + N} - \delta_{N_p} N_p. \tag{S7d}$$

The equilibria of System (S7) take the form of:

$$\begin{aligned} T^* &= \frac{\alpha_N \Pi_T N^* - \delta_{T_p} \Pi_N (K_N + N^*)}{\alpha_N \beta N^{*2}}, \\ T_p^* &= \frac{\Pi_T - \beta N^* T^*}{\delta_{T_p}} = \frac{\Pi_N (K_N + N^*)}{\alpha_N N^*}, \\ N_p^* &= \frac{\Pi_N}{\delta_{N_p}}, \end{aligned} \tag{S8}$$

where N^* is a positive solution of the following polynomial of order 5 in N^*:

$$\begin{aligned} &\frac{\Pi_N (\alpha_{T_p} + \Pi_T)(K_N + N^*) + \alpha_N K_{T_p} \Pi_T N^*}{\alpha_N K_{T_p} N^* + \Pi_N (K_N + N^*)} \\ =&\frac{(\alpha_N \Pi_T N^* - \delta_{T_p} \Pi_N)(K_N + N^*)(\beta K_T N^{*2} + \Pi_T N^* - \frac{\delta_{T_p} \Pi_N}{\alpha_N}(K_N + N^*) + \alpha_T)}{\alpha_N \beta K_T N^{*2} + \alpha_N \Pi_T N^* - \delta_{T_p} \Pi_N (K_N + N^*)}, \end{aligned} \tag{S9}$$

which has between 1 and 3 positive real roots from Descartes' rule of signs. Furthermore, the following condition is required for the positivity of equilibrium components defined in (S8):

$$N^* > \frac{\delta_{T_p}\Pi_N K_N}{\alpha_N \Pi_T - \delta_{T_p}\Pi_N} > 0 \tag{S10}$$

Using linearization and Routh-Hurwitz criteria, an equilibrium is locally asymptotically stable if N^* satisfies the following condition:

$$\begin{aligned}&\frac{\alpha_T K_T \alpha_N \beta N^*(\delta_{T_p}\Pi_N(K_N + 2N^*) - \alpha_N \Pi_T N^*)}{(\alpha_N \beta K_T N^{*2} + \alpha_N \Pi_T N^* - \delta_{T_p}\Pi_N(K_N + N^*))^2} \\ &+\Pi_N \left(\frac{\alpha_{T_p} K_{T_p} \alpha_N N^*}{(K_{T_p}\alpha_N N^* + \Pi_N(K_N + N^*))^2} + \frac{\delta_{T_p}}{\alpha_N N^*} \right) > 0\end{aligned} \tag{S11}$$

Numerically, when there exist positive equilibria, at least one is locally asymptotically stable.

S2.1.2 NMT1 and mTOR dynamics with rapamycin

We assumed in this section that the concentration of rapamycin R remains constant in the cell for the period considered. These models are characterized by a reversible or irreversible rapamycin binding and the presence or absence of feedback. When possible (reversible binding and no feedback) we consider also a constant total mTOR instead of an explicit synthesis and degradation.

S2.1.2.1 Without feedback – Models NTRr, NTtRr and NTRi

S2.1.2.1.1 Reversible effect of rapamycin and synthesis/degradation of mTOR – Model NTRr Adding a reversible effect of rapamycin to Model NT (System (S1)) leads to Model NTRr:

$$\frac{\mathrm{d}T}{\mathrm{d}t} = -\frac{\alpha_T T}{K_T + T} + \frac{\alpha_{T_p} T_p}{K_{T_p} + T_p} - \gamma R T + \kappa R_c + \Pi_T, \tag{S12a}$$

$$\frac{\mathrm{d}T_p}{\mathrm{d}t} = \frac{\alpha_T T}{K_T + T} - \frac{\alpha_{T_p} T_p}{K_{T_p} + T_p} - \delta_{T_p} T_p, \tag{S12b}$$

$$\frac{\mathrm{d}N}{\mathrm{d}t} = -\frac{\alpha_N T_p N}{K_N + N} + \Pi_N, \tag{S12c}$$

$$\frac{\mathrm{d}N_p}{\mathrm{d}t} = \frac{\alpha_N T_p N}{K_N + N} - \delta_{N_p} N_p, \tag{S12d}$$

$$\frac{\mathrm{d}R_c}{\mathrm{d}t} = \gamma R T - \kappa R_c. \tag{S12e}$$

If the condition of existence (S3) is fulfilled, System (S12) has a unique positive equilibrium $(T^*, T_p^*, N^*, N_p^*, R_c^*)$, expressed as follows

$$\begin{aligned} T^* &= \frac{\Pi_T K_T \left(1 + \frac{\alpha_{T_p}}{\delta_{T_p} K_{T_p} + \Pi_T}\right)}{\alpha_T - \Pi_T \left(1 + \frac{\alpha_{T_p}}{\delta_{T_p} K_{T_p} + \Pi_T}\right)} \\ T_p^* &= \frac{\Pi_T}{\delta_{T_p}}, \\ N^* &= \frac{\Pi_N K_N \delta_{T_p}}{\alpha_N \Pi_T - \Pi_N \delta_{T_p}}, \\ N_p^* &= \frac{\Pi_N}{\delta_{N_p}}, \\ R_c^* &= \frac{\gamma R}{\kappa} T^*. \end{aligned} \tag{S13}$$

After linearization of System (S12), the equilibrium is shown to be locally asymptotically stable when it exists.

S2.1.2.1.2 Reversible effect and constant total mTOR T_t – Model NTtRr

$$\frac{\mathrm{d}T}{\mathrm{d}t} = -\frac{\alpha_T T}{K_T + T} + \frac{\alpha_{T_p}(T_t - T - R_c)}{K_{T_p} + T_t - T - R_c} - \gamma R T + \kappa R_c, \tag{S14a}$$

$$\frac{\mathrm{d}N}{\mathrm{d}t} = -\frac{\alpha_N (T_t - T - R_c) N}{K_N + N} + \Pi_N, \tag{S14b}$$

$$\frac{\mathrm{d}N_p}{\mathrm{d}t} = \frac{\alpha_N (T_t - T - R_c) N}{K_N + N} - \delta_{N_p} N_p, \tag{S14c}$$

$$\frac{\mathrm{d}R_c}{\mathrm{d}t} = \gamma R T - \kappa R_c. \tag{S14d}$$

System (S14) has a unique positive equilibrium $(T^*, T_p^*, N^*, N_p^*, R_c^*)$ if $T^* < \frac{\alpha_N T_t - \Pi_N}{\alpha_N(1+\gamma R/\kappa)}$ where

$$T^* = \begin{cases} \dfrac{-B - \sqrt{B^2 - 4AC}}{2A} & \text{if } \alpha_T - \alpha_{T_p} \neq 0, \\ \dfrac{-C}{B} & \text{if } \alpha_T - \alpha_{T_p} = 0, \end{cases} \tag{S15}$$

with $A = (\alpha_T - \alpha_{T_p})\left(1 + \frac{\gamma R}{\kappa}\right)$, $B = -(\alpha_T - \alpha_{T_p})T_t - \alpha_T K_{T_p} - \alpha_{T_p} K_T \left(1 + \frac{\gamma R}{\kappa}\right)$ and $C = \alpha_{T_p} K_T T_t$. The other components of equilibrium are

$$\begin{aligned} T_p^* &= T_t - T^* - R_c^* = T_t - \left(1 + \frac{\gamma R}{\kappa}\right) T^*, \\ N^* &= \frac{\Pi_N K_N}{\alpha_N T_p^* - \Pi_N}, \\ N_p^* &= \frac{\Pi_N}{\delta_{N_p}}, \\ R_c^* &= \frac{\gamma R}{\kappa} T^*. \end{aligned} \tag{S16}$$

Using the linearization of System (S14), it has been shown that the unique positive equilibrium is locally asymptotically stable, when it exists.

S2.1.2.1.3 Irreversible effect – Model NTRi In order to consider irreversible binding with rapamycin without depleting all mTOR, we included production and degradation in the mTOR subsystem, which were neglected in the previous model.

$$\frac{\mathrm{d}T}{\mathrm{d}t} = -\frac{\alpha_T T}{K_T + T} + \frac{\alpha_{T_p} T_p}{K_{T_p} + T_p} - \gamma R T + \Pi_T, \tag{S17a}$$

$$\frac{\mathrm{d}T_p}{\mathrm{d}t} = \frac{\alpha_T T}{K_T + T} - \frac{\alpha_{T_p} T_p}{K_{T_p} + T_p} - \delta_{T_p} T_p, \tag{S17b}$$

$$\frac{\mathrm{d}N}{\mathrm{d}t} = -\frac{\alpha_N T_p N}{K_N + N} + \Pi_N, \tag{S17c}$$

$$\frac{\mathrm{d}N_p}{\mathrm{d}t} = \frac{\alpha_N T_p N}{K_N + N} - \delta_{N_p} N_p, \tag{S17d}$$

$$\frac{\mathrm{d}R_c}{\mathrm{d}t} = \gamma R T - \delta_{R_c} R_c. \tag{S17e}$$

An equilibrium $(T^*, T_p^*, N^*, N_p^*, R_c^*)$ of System (S17) is expressed as follows:

$$\begin{aligned} T_p^* &= \frac{\Pi_T - \gamma R T^*}{\delta_{T_p}}, \\ N^* &= \frac{\Pi_N K_N}{\alpha_N T_p^* - \Pi_N} = \frac{\Pi_N \delta_{T_p} K_N}{\alpha_N(\Pi_T - \gamma R T^*) - \delta_{T_p}\Pi_N}, \\ N_p^* &= \frac{\Pi_N}{\delta_{N_p}}, \\ R_c^* &= \frac{\gamma R}{\delta_{R_c}} T^*, \end{aligned} \tag{S18}$$

where T^* is a positive root of the following function

$$f(T^*) = -\frac{\alpha_T T^*}{K_T + T^*} + \frac{\alpha_{T_p}(\Pi_T - \gamma R T^*)}{K_{T_p}\delta_{T_p} + (\Pi_T - \gamma R T^*)} - \gamma R T^* + \Pi_T = 0. \tag{S19}$$

The roots of $f(T^*)$ are not explicitly found; however, it can be shown that there exists a unique T^* between 0 and $\frac{\Pi_T}{\gamma R}$ that preserves the positivity of T_p^*. Additionally, to ensure the positivity of N^* a more restrictive condition is required:

$$T^* < \frac{\Pi_T}{\gamma R} - \frac{\Pi_N \delta_{T_p}}{\alpha_N \gamma R}. \tag{S20}$$

It has been shown after linearization that, when it exists, this equilibrium is locally asymptotically stable.

S2.1.2.2 With feedback – Models NTRrf and NTRif In the following models, we assumed that there is a feedback regulation of mTOR by NMT1, modelled by a degradation of mTOR depending on the concentration of NMT1.

S2.1.2.2.1 Reversible effect – Model NTRrf Adding a reversible effect of rapamycin to Model NTf (System (S7)) leads to Model NTRrf:

$$\frac{\mathrm{d}T}{\mathrm{d}t} = -\frac{\alpha_T T}{K_T + T} + \frac{\alpha_{T_p} T_p}{K_{T_p} + T_p} - \gamma R T + \kappa R_c + \Pi_T - \beta N T, \tag{S21a}$$

$$\frac{\mathrm{d}T_p}{\mathrm{d}t} = \frac{\alpha_T T}{K_T + T} - \frac{\alpha_{T_p} T_p}{K_{T_p} + T_p} - \delta_{T_p} T_p, \tag{S21b}$$

$$\frac{\mathrm{d}N}{\mathrm{d}t} = -\frac{\alpha_N T_p N}{K_N + N} + \Pi_N, \tag{S21c}$$

$$\frac{\mathrm{d}N_p}{\mathrm{d}t} = \frac{\alpha_N T_p N}{K_N + N} - \delta_{N_p} N_p, \tag{S21d}$$

$$\frac{\mathrm{d}R_c}{\mathrm{d}t} = \gamma R T - \kappa R_c. \tag{S21e}$$

When the existence condition (S10) is satisfied, there are positive equilibria for System (S21) expressed as follows:

$$\begin{aligned} T^* &= \frac{\alpha_N \Pi_T N^* - \delta_{T_p}\Pi_N (K_N + N^*)}{\alpha_N \beta N^{*2}}, \\ T_p^* &= \frac{\Pi_T - \beta N^* T^*}{\delta_{T_p}} = \frac{\Pi_N (K_N + N^*)}{\alpha_N N^*}, \\ N_p^* &= \frac{\Pi_N}{\delta_{N_p}}, \\ R_c^* &= \frac{\gamma R T^*}{\kappa} = \gamma R \frac{\alpha_N \Pi_T N^* - \delta_{T_p}\Pi_N (K_N + N^*)}{\kappa \alpha_N \beta N^{*2}}, \end{aligned} \tag{S22}$$

where N^* is a positive root of the following equation:

$$
\begin{aligned}
&-\alpha_T \frac{\alpha_N \Pi_T N^* - \delta_{T_p} \Pi_N (K_N + N^*)}{\alpha_N \beta N^{*2}} \left(K_{T_p}\delta_{T_p} + \Pi_T - \frac{\alpha_N \Pi_T N^* - \delta_{T_p} \Pi_N (K_N + N^*)}{\alpha_N N^*} \right) \\
&\quad + \left(K_T + \frac{\alpha_N \Pi_T N^* - \delta_{T_p} \Pi_N (K_N + N^*)}{\alpha_N \beta N^{*2}} \right) \left(\Pi_T - \frac{\alpha_N \Pi_T N^* - \delta_{T_p} \Pi_N (K_N + N^*)}{\alpha_N N^*} \right) \\
&\qquad \times \left(K_{T_p}\delta_{T_p} + \Pi_T - \frac{\alpha_N \Pi_T N^* - \delta_{T_p} \Pi_N (K_N + N^*)}{\alpha_N N^*} + \alpha_{T_p} \right) = 0.
\end{aligned} \tag{S23}
$$

Using linearization and Routh-Hurwitz criteria, a positive equilibrium is locally asymptotically stable if $a_4 > 0$ and $a_1 a_2 a_3 > a_3^2 + a_1^2 a_4$, with

$$
\begin{aligned}
a_1 &= 1 + \kappa + \beta N^* + \frac{\alpha_{T_p} K_{T_p}}{(K_{T_p} + T_p^*)^2} + \delta_{T_p} + \frac{K_N \Pi_N}{N^*(K_N + N^*)} + \frac{\alpha_T K_T}{(K_T + T^*)^2}, \\
a_2 &= (\gamma R + \beta N^*)(B + \delta_{T_p} + D) + \kappa(B + \delta_{T_p} + \beta N^* + A + D) + D(B + \delta_{T_p}) + A(D + \delta_{T_p}), \\
a_3 &= D(B + \delta_{T_p})(\gamma R + \kappa + \beta N^*) + \kappa(\beta N^*(B + \delta_{T_p} + D) + A(D + \delta_{T_p})) + AD\delta_{T_p} - AC\beta T^*, \\
a_4 &= \kappa(D(\beta N^*(B + \delta_{T_p}) + A\delta_{T_p}) - AC\beta T^*,
\end{aligned} \tag{S24}
$$

where $A = \dfrac{\alpha_T K_T}{(K_T + T^*)^2}$, $B = \dfrac{\alpha_{T_p} K_{T_p}}{(K_{T_p} + T_p^*)^2}$, $C = \dfrac{\alpha_N K_N}{K_N + N^*}$ and $D = \dfrac{K_N \Pi_N}{N^*(K_N + N^*)}$.

S2.1.2.2.2 Irreversible effect – Model NTRif

$$
\frac{\mathrm{d}T}{\mathrm{d}t} = -\frac{\alpha_T T}{K_T + T} + \frac{\alpha_{T_p} T_p}{K_{T_p} + T_p} - \gamma RT + \Pi_T - \beta NT, \tag{S25a}
$$

$$
\frac{\mathrm{d}T_p}{\mathrm{d}t} = \frac{\alpha_T T}{K_T + T} - \frac{\alpha_{T_p} T_p}{K_{T_p} + T_p} - \delta_{T_p} T_p, \tag{S25b}
$$

$$
\frac{\mathrm{d}N}{\mathrm{d}t} = -\frac{\alpha_N T_p N}{K_N + N} + \Pi_N, \tag{S25c}
$$

$$
\frac{\mathrm{d}N_p}{\mathrm{d}t} = \frac{\alpha_N T_p N}{K_N + N} - \delta_{N_p} N_p, \tag{S25d}
$$

$$
\frac{\mathrm{d}R_c}{\mathrm{d}t} = \gamma RT - \delta_{R_c} R_c. \tag{S25e}
$$

When the existence condition (S10) is satisfied, it can be shown that System (S25) has positive equilibria

$$
\begin{aligned}
T^* &= \frac{\alpha_N \Pi_T N^* - \delta_{T_p} \Pi_N (K_N + N^*)}{\alpha_N (\gamma R + \beta N^*) N^*}, \\
T_p^* &= \frac{\Pi_N (K_N + N^*)}{\alpha_N N^*}, \\
N_p^* &= \frac{\Pi_N}{\delta_{N_p}}, \\
R_c^* &= \frac{\gamma R T^*}{\delta_{R_c}},
\end{aligned} \tag{S26}
$$

where N^* is a positive solution of the following equation:

$$
\begin{aligned}
&\frac{\alpha_N \Pi_T N^* - \delta_{T_p} \Pi_N (K_N + N^*)}{\alpha_N N^*} + \frac{\alpha_T (\alpha_N \Pi_T N^* - \delta_{T_p} \Pi_N (K_N + N^*))}{\alpha_N K_T (\gamma R + \beta N^*) N^* + \alpha_N \Pi_T N^* - \delta_{T_p} \Pi_N (K_N + N^*)} \\
=&\Pi_T + \frac{\alpha_{T_p} \Pi_N (K_N + N^*)}{\alpha_N K_{T_p} N^* + \Pi_N (K_N + N^*)}.
\end{aligned} \tag{S27}
$$

Using linearization and Routh-Hurwitz criteria, an equilibrium is locally asymptotically stable

if N^* verifies the condition

$$\frac{\delta_{T_p}\Pi_N(\gamma R+\beta N^*)}{\alpha_N N^*}\left(1+\frac{\alpha_T K_T\alpha_N^2 N^{*2}(\gamma R+\beta N)}{(\alpha_N K_T(\gamma R+\beta N^*)N^*+\alpha_N\Pi_T N^*-\delta_{T_p}\Pi_N(K_N+N^*))^2}\right)>$$
$$\frac{\beta\alpha_T K_T\alpha_N N^*(K_N+N^*)(\alpha_N\Pi_T N^*-\delta_{T_p}\Pi_N(K_N+N^*))}{(\alpha_N K_T(\gamma R+\beta N^*)N^*+\alpha_N\Pi_T N^*-\delta_{T_p}\Pi_N(K_N+N^*))^2}. \tag{S28}$$

S2.1.3 Summary

A summary of local stability analysis results is given in Table S1.

Model	Rapamycin effect	Feedback	Existence	LAS	positive equilibria
NT (S1)	no	no	(S3)	WE	1 LAS
NTt (S4)	no	no	$T_p^* > \frac{\Pi_N}{\alpha_N}$	WE	1 LAS
NTf (S7)	no	yes	(S10)	(S11)	1 LAS & 1 unstable
NTRr (S12)	reversible	no	(S3)	WE	1 LAS
NTtRr (S14)	reversible	no	$T^* < \frac{\alpha_N T_t-\Pi_N}{\alpha_N(1+\gamma R/\kappa)}$	WE	1 LAS
NTRrf (S21)	reversible	yes	(S10)	(S24)	1 LAS & 1 unstable
NTRi (S17)	irreversible	no	(S20)	WE	1 LAS
NTRif (S25)	irreversible	yes	(S10)	(S28)	1 LAS & 1 unstable

Table S1: Summary of the models assumptions and properties, conditions of existence of positive equilibrium and local asymptotic stabilities. WE = when the equilibrium exists.

Similar analyses have also been performed when considering the dephosphorylation of p-NMT1 in System (1), as described in System (S29), and the asymptotic properties of the models remain the same (results not shown).

$$\frac{\mathrm{d}T}{\mathrm{d}t}=-\frac{\alpha_T T}{K_T+T}+\frac{\alpha_{T_p}T_p}{K_{T_p}+T_p}+\Pi_T+f(T,N)+g(T,R_c), \tag{S29a}$$

$$\frac{\mathrm{d}T_p}{\mathrm{d}t}=\frac{\alpha_T T}{K_T+T}-\frac{\alpha_{T_p}T_p}{K_{T_p}+T_p}-\delta_{T_p}T_p, \tag{S29b}$$

$$\frac{\mathrm{d}N}{\mathrm{d}t}=-\frac{\alpha_N T_p N}{K_N+N}+\overbrace{\frac{\alpha_{N_p}N_p}{K_{N_p}+N_p}}^{\text{pNMT1 dephosphorylation}}+\Pi_N, \tag{S29c}$$

$$\frac{\mathrm{d}N_p}{\mathrm{d}t}=\frac{\alpha_N T_p N}{K_N+N}-\overbrace{\frac{\alpha_{N_p}N_p}{K_{N_p}+N_p}}^{\text{pNMT1 dephosphorylation}}-\delta_{N_p}N_p, \tag{S29d}$$

$$\frac{\mathrm{d}R_c}{\mathrm{d}t}=h(T,R_c). \tag{S29e}$$

S2.2 Numerical results

S2.2.1 Model calibration and selection

We compared the model output to the following experimental data over time: total mTOR $T_{tot} = T + T_p$ or $T_{tot} = T + T_p + R_c$, proportion of phosphorylated mTOR, T_p/T_{tot}, and total NMT1, $N + N_p$. NMT1 and mTOR experimental values are normalized against the housekeeping protein β-actin.

We computed the residual sum of squares RSS between experimental data points and model outputs, and minimized this function to find the best set of parameter values to fit experimental data. The best set is also called the nominal set. The optimization problem is solved using a genetic algorithm repeated multiple times. All the optimization procedure is performed using Matlab R2016b.

S2.2.1.1 Models without rapamycin – Control The best scenario for all datasets corresponds to model NTt, as highlighted in Table 2. In Figure S4, the best fit for models NT, NTt and NTf on all four datasets is displayed.

Using the sets of parameter values with the lowest RSS for model NTt (lower than $\text{RSS}_{min} \times 1.25$), we obtained a distribution of values for each parameter (see Figure S6). The estimated values of parameters is of the same order of magnitude in all datasets (see Figure S6), but some differences between datasets can be observed and result from experimental data (the proportion of p-mTOR is higher for dataset 2 while total NMT1 is higher for dataset 1). In particular, one may note that the estimation for T_t, corresponding to the total amount of mTOR, is different for each dataset and has a low variability within each dataset. Parameters responsible for the dynamics of NMT1, in particular Π_N, tend to be lower for dataset 2, due to the low value of total NMT1 in this dataset.

S2.2.1.2 Models with rapamycin - Perturbation The best scenario with rapamycin corresponds to an absence of feedback regulation of mTOR (similarly to the control results) and a reversible binding with rapamycin, as highlighted in Table 2. Model NTtRr, with a constant total mTOR, is the best model for datasets 2 and 3, with an Akaike weight above 0.9. Figure S5 illustrates the best fit for models NTRr, NTtRr, NTRi,NTRrf and NTRif on datasets 1 to 4. Moreover, using the sets of parameter values with the lowest RSS for model NTtRr (lower than $\text{RSS}_{min} \times 1.25$), we obtain a distribution of values for each parameter (see Figure S6).

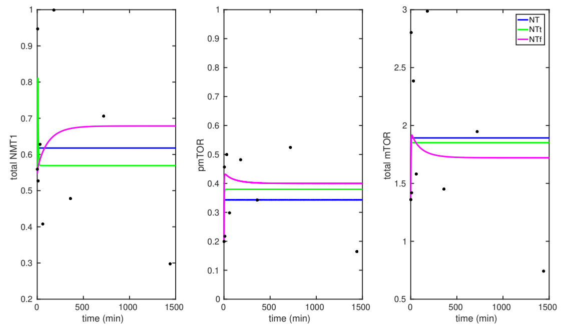

(a) Dataset 1

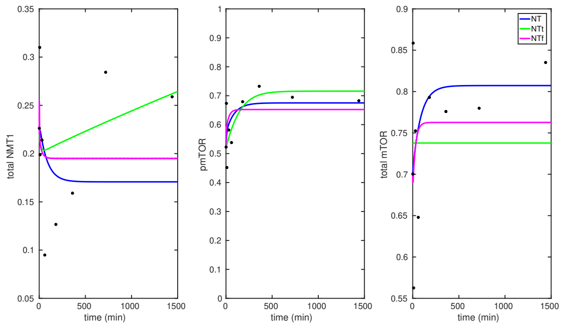

(b) Dataset 2

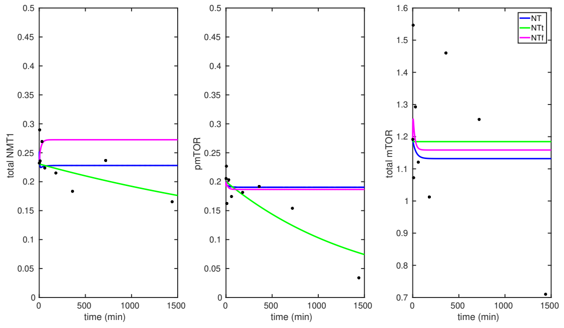

(c) Dataset 3

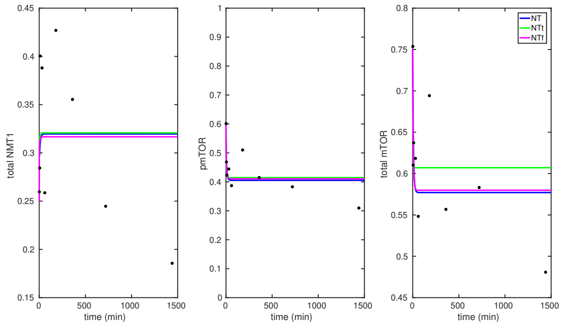

(d) Dataset 4

Figure S4: Best fits for models without rapamycin NT, NTt and NTf for dataset 1, 2, 3 and 4.

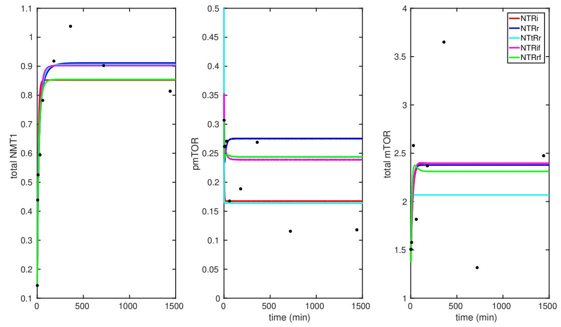

(a) Dataset 1

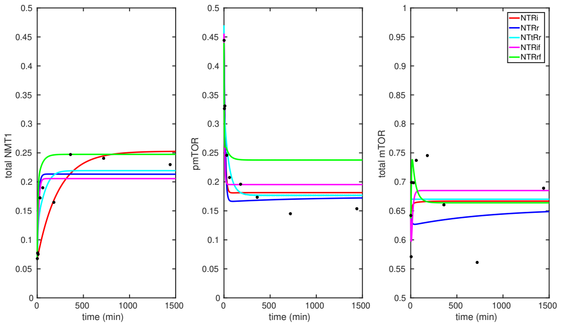

(b) Dataset 2

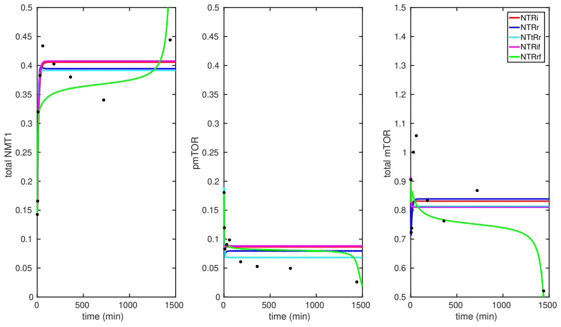

(c) Dataset 3

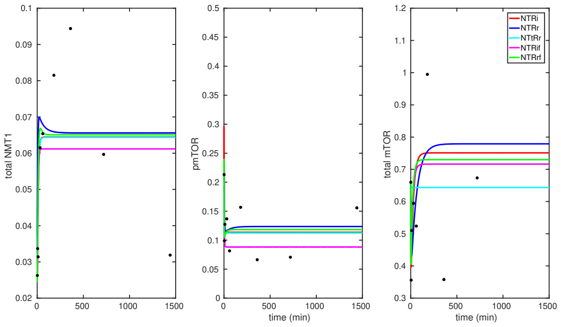

(d) Dataset 4

Figure S5: Best fits for models with rapamycin NTRr, NTtRr, NTRi, NTRrf and NTRif for datasets 1, 2, 3 and 4.

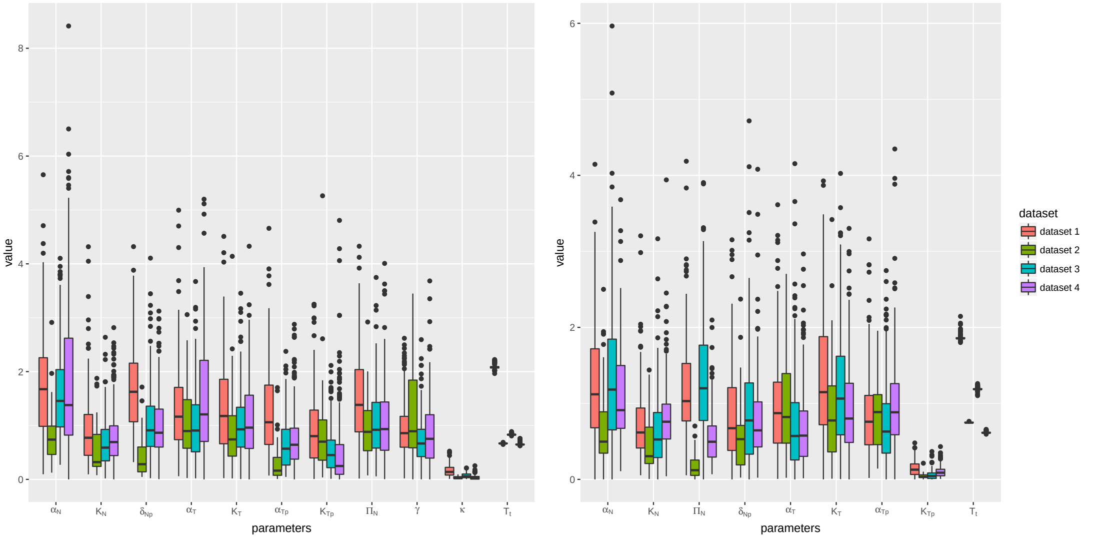

(a) With rapamycin – Model NTtRr

(b) Without rapamycin – Model NTt

Figure S6: Boxplot of the best sets of parameter values for model NTtRr (a) and model NTt (b), for datasets 1 to 4. The best sets of parameter values verify RSS $< 1.25\text{RSS}_{min}$.

S2.2.2 Robustness of the models

Random sets of parameter values were generated by using a Uniform distribution between 0.9 and 1.1 times the values of the nominal parameter set. This allowed us to assess the robustness of models to small variations of the nominal set of parameters. On Figures S7 and S8, model outputs obtained with these random sets were used to compute an envelope whose boundaries are the minimal and maximal values of the simulations. One may note that the ratio p-mTOR/total mTOR is almost not impacted by small variations in parameter values. Total mTOR and NMT1 are slightly affected.

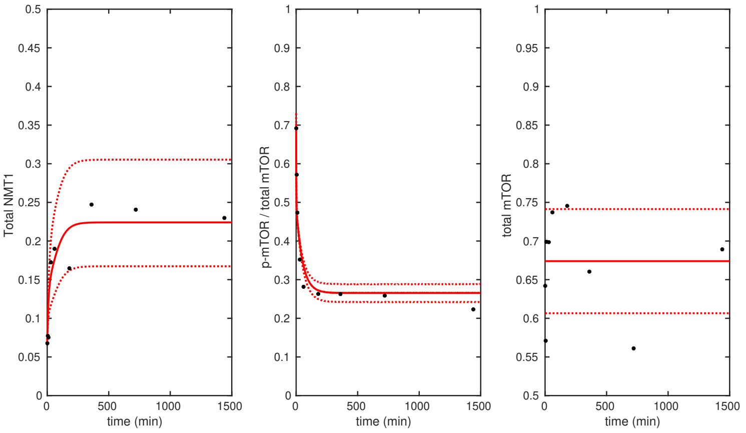

(a) Dataset 2

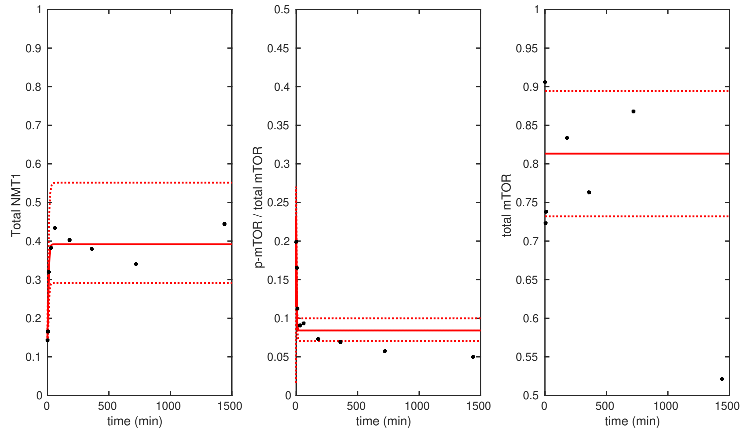

(b) Dataset 3

Figure S7: Best fit for model NTtRr for datasets 2 and 3 with rapamycin (solid line), with the envelope (dotted lines) corresponding to a variation of ±10% of the nominal parameter values (best set of parameters).

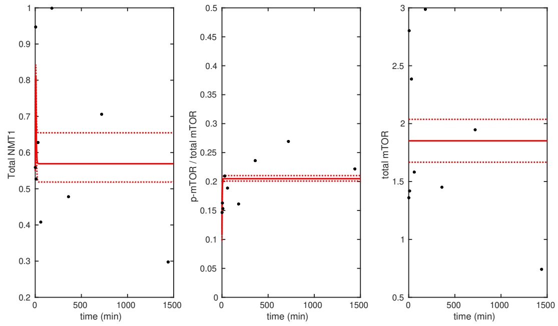

(a) Dataset 1

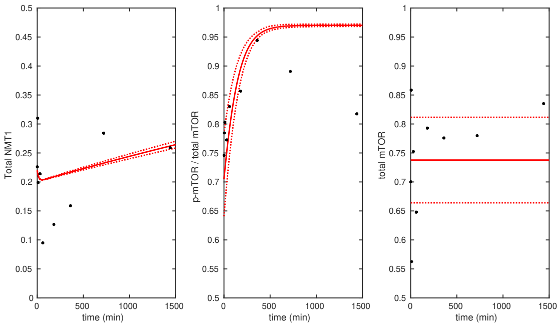

(b) Dataset 2

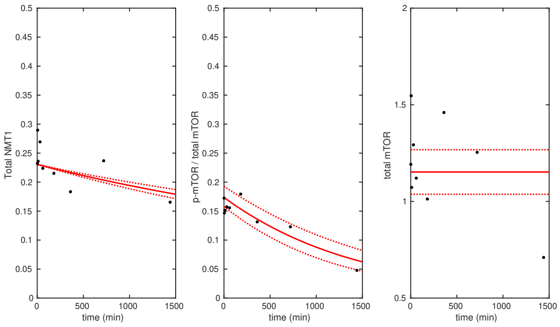

(c) Dataset 3

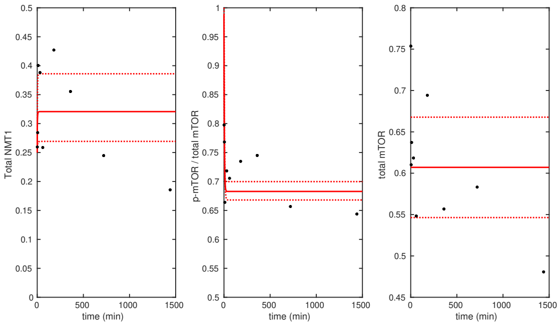

(d) Dataset 4

Figure S8: Best fit for model NTt for datasets 1, 2, 3 and 4 without rapamycin (solid line), with the envelope (dotted lines) corresponding to a variation of ±10% of the nominal parameter values (best set of parameters).

S2.2.3 Application to additional cell line (T47D)

Models NTt and NTtRr were fitted to experimental data obtained for T47D cell line with and without rapamycin. Parameter values are in the range of parameter values obtained for MCF7 cell line and the models are able to reproduce experimental data (see Figures S9 and S10).

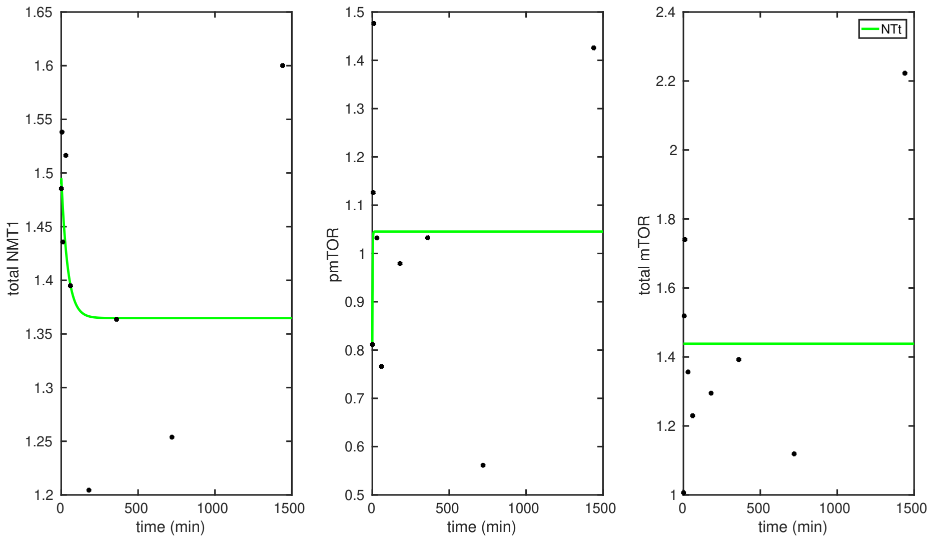

Figure S9: Best fit for model NTt for T47D data without rapamycin.

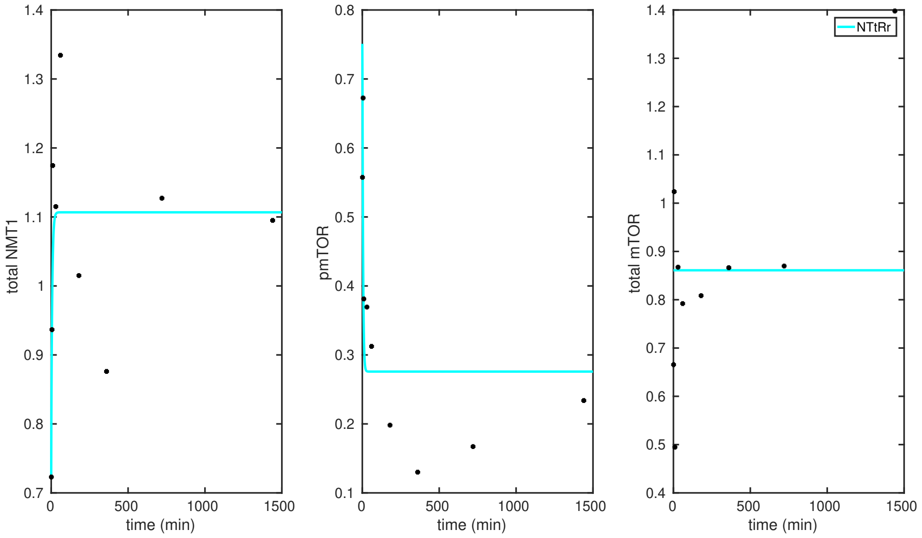

Figure S10: Best fit for model NTtRr for T47D data with rapamycin.

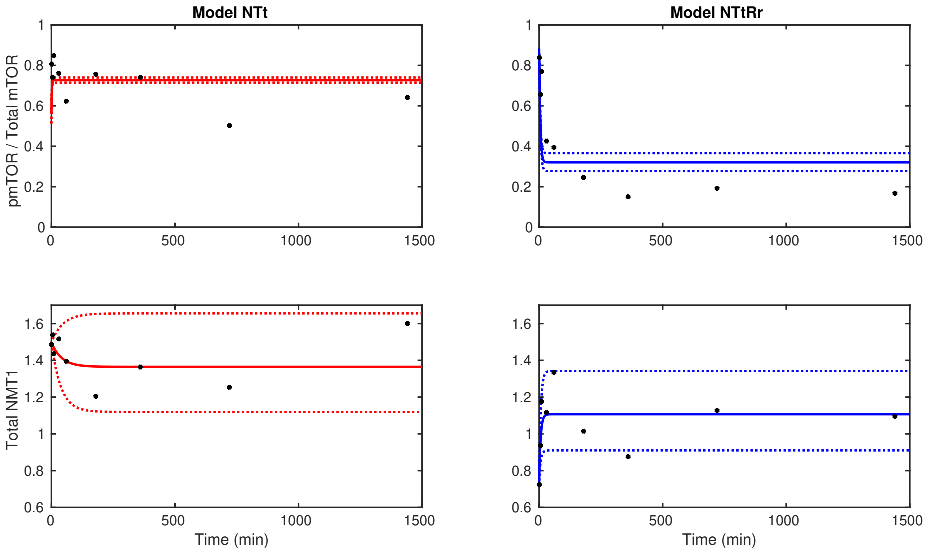

Figure S11: Best fit for models NTt (without rapamycin, on the left) and NTtRr (with rapamycin, on the right) for T47D data (solid line), with the envelope (dotted lines) corresponding to a variation of ±10% of the nominal parameter values (best set of parameters).

S2.2.4 Global sensitivity analysis

We used the LHS-PRCC method (Partial Rank Correlation Coefficients with Latin Hypercube Sampling) to compute the global sensitivity of the models.

S2.2.4.1 Sampling We generated sets of parameter values using Latin Hypercube sampling (LHS), with a Uniform distribution for each parameter. Estimates of parameter values were used to set the sampling intervals. As listed in Table S1, the existence of a positive equilibrium depends on parameter values, thus we selected only the sets of parameter values leading to a biologically plausible situation, as similarly done in Marino et al. (2008). Thus we performed the PRCC analysis on the relevant sets of parameters. Only 10 to 15% of the sets of parameters validate the conditions. Hence, we obtained a posteriori distributions of parameter values corresponding to the valid sets of parameters. Most a posteriori distributions were not uniform anymore, with major exclusions of extremal values for some parameters (see Figure S12).

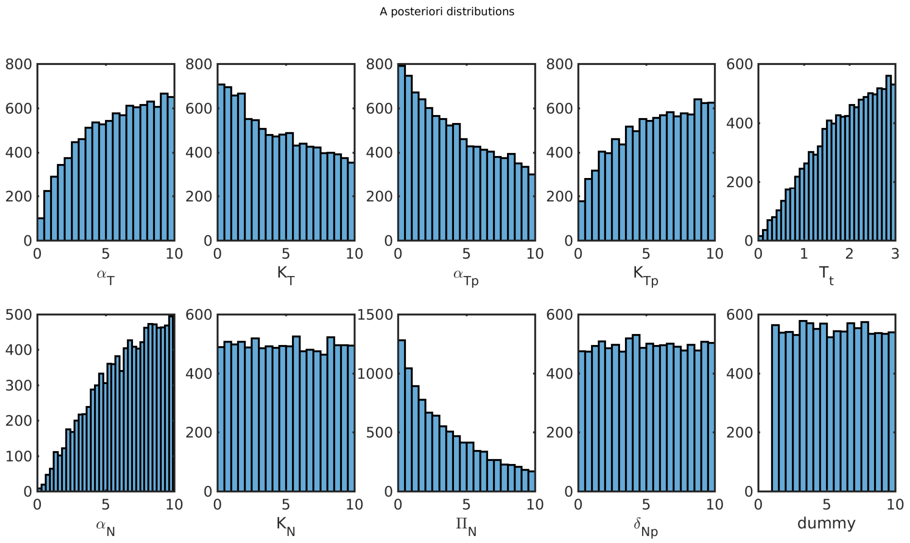

Figure S12: A posteriori distributions of parameters for model NTt starting from a Uniform distribution.

S2.2.4.2 PRCC analysis Using the sets of parameters selected as described above, we simulated the models until time $t = 2880$ minutes to obtain the steady state value of variables N, N_p, T or T_p. Results of PRCC analysis are only shown for the best models with and without rapamycin, namely models NTt and NTtRr (see Figure S13). The PRCC analysis is significant if the coefficient is different from 0. In Figure S13, three significativity levels are displayed. One may note that the PRCC associated to the dummy parameter is not significantly different from 0, as expected.

All parameters have a significant impact on a least one of the variables. One may note that the coefficients for N and N_p are similar in models with and without rapamycin, while, as expected, coefficients for T (mTOR) and T_p (p-mTOR) have opposite values.

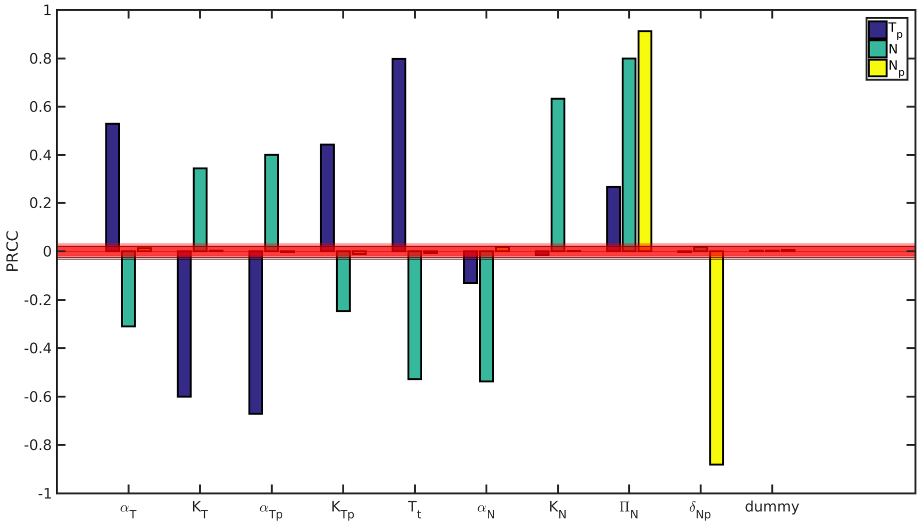

(a) Model NTt

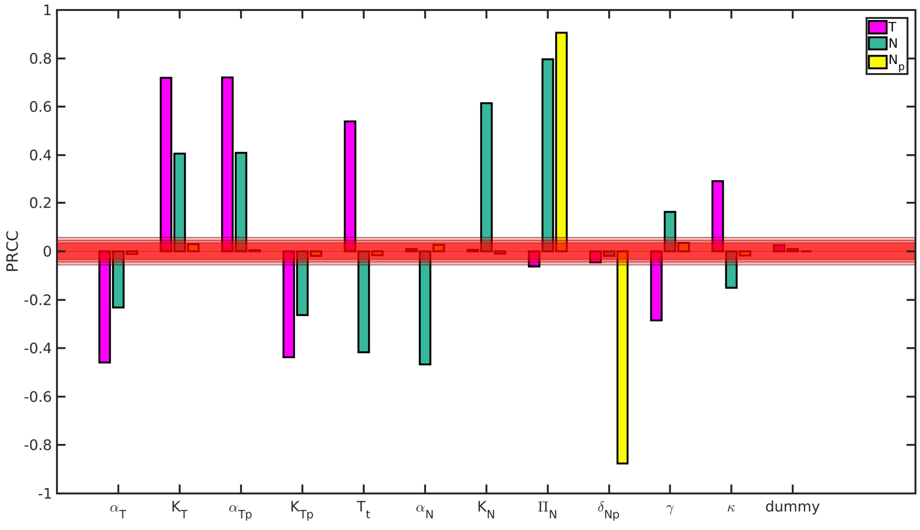

(b) Model NTtRr

Figure S13: Global sensitivities obtained by LHS-PRCC for models NTt and NTtRr. The red areas, from lighter to darker colors, indicate three significativity levels 0.001, 0.01 and 0.05.
